# Genetic dissection of the polyoxin building block-carbamoylpolyoxamic acid biosynthesis revealing the “pathway redundancy” in metabolic networks

**DOI:** 10.1186/1475-2859-12-121

**Published:** 2013-12-07

**Authors:** Wenqing Chen, Daofeng Dai, Changchun Wang, Tingting Huang, Lipeng Zhai, Zixin Deng

**Affiliations:** 1 Key Laboratory of Combinatorial Biosynthesis and Drug Discovery, Ministry of Education, and School of Pharmaceutical Sciences, Wuhan University, 185 East Lake Road, Wuhan 430071, P.R. China; 2 State Key Laboratory of Bioreactor Engineering, East China University of Science and Technology, Shanghai 200237, China; 3 State Key Laboratory of Microbial Metabolism, and School of Life Sciences & Biotechnology, Shanghai Jiao Tong University, Shanghai 200030, China; 4 Department of Chemical and Biomolecular Engineering, University of California, Los Angeles, California 90095, USA

**Keywords:** Polyoxin, Building block, Carbamoylpolyoxamic acid, Pathway redundancy, Metabolic networks

## Abstract

**Background:**

Polyoxin, a peptidyl nucleoside antibiotic, consists of three building blocks including a nucleoside skeleton, polyoximic acid (POIA), and carbamoylpolyoxamic acid (CPOAA), however, little is known about the “pathway redundancy” of the metabolic networks directing the CPOAA biosynthesis in the cell factories of the polyoxin producer.

**Results:**

Here we report the genetic characterization of CPOAA biosynthesis with revealing a “pathway redundancy” in metabolic networks. Independent mutation of the four genes (*polL-N* and *polP*) directly resulted in the accumulation of polyoxin I, suggesting their positive roles for CPOAA biosynthesis. Moreover, the individual mutant of *polN* and *polP* also partially retains polyoxin production, suggesting the existence of the alternative homologs substituting their functional roles.

**Conclusions:**

It is unveiled that *argA* and *argB* in L-arginine biosynthetic pathway contributed to the “pathway redundancy”, more interestingly, *argB* in *S. cacaoi* is indispensible for both polyoxin production and L-arginine biosynthesis. These data should provide an example for the research on the “pathway redundancy” in metabolic networks, and lay a solid foundation for targeted enhancement of polyoxin production with synthetic biology strategies.

## Introduction

**Figure 1:**
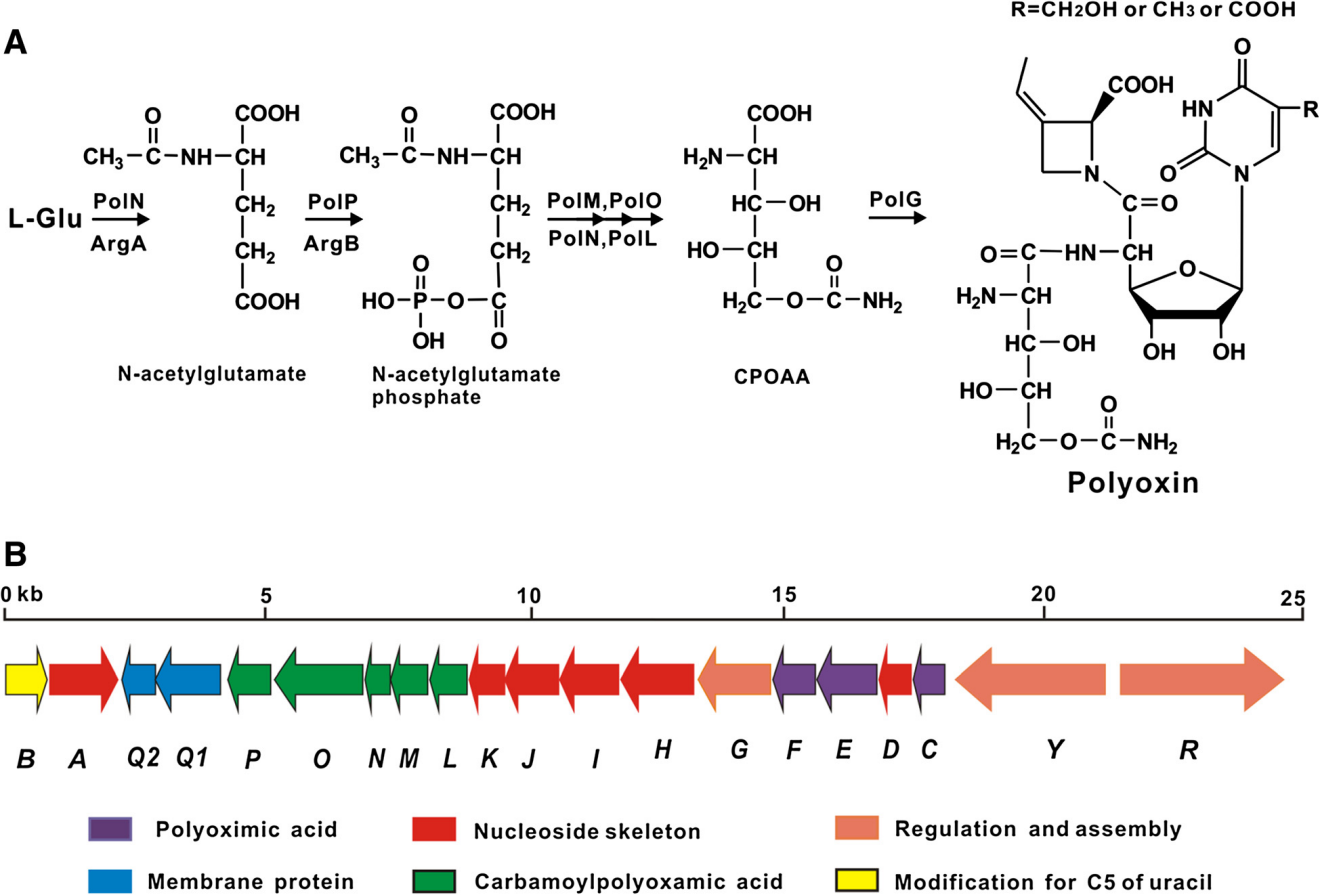
**The proposed CPOAA biosynthetic pathway (A) and Genetic organization of the polyoxin biosynthetic gene cluster (B).** The initial two steps involved in CPOAA biosynthesis are overlapped with those of L-Arginine pathway.

Nucleoside antibiotics, a family of important natural products with microbial origin, have become research focus in recent years for their diverse bioactivities and promising application potentials [1,2]; usually, this family of antibiotics harbors intricate structural features by modification of nucleosides or nucleotides [2]. Polyoxins (Figure 1A), a group of structurally-related nucleoside antibiotics produced by *Streptomyces cacaoi* var. *asoensis* (*S. cacaoi* hereafter) [3-5] and *Streptomyces aureochromogenes *[6], exhibit potent bioactivity against phytopathoengenic fungi [2,7]. As the first nucleoside antibiotic targeting fungal cell wall biosynthesis, polyoxin displays similar structural features to that of UDP-N-acetylglucosamine, a substrate for chitin biosynthesis, and thus it functions as a powerful competitive inhibitor of chitin synthetase [7,8].

The structure of polyoxin is composed of three building blocks including a nucleoside skeleton, polyoximic acid (POIA) and carbamoylpolyoxamic acid. Previous studies with feeding experiments have demonstrated that the three building blocks were individually originated from uridine (or UMP), L-isoleucine and L-glutamate [2]. Previously, the polyoxin biosynthetic gene cluster was cloned and well characterized, and the entire polyoxin gene cluster was revealed to consist of 20 genes (Figure 1B), of them, three genes (*polF*, *polC* and *polE*) were proposed to be involved in POIA biosynthesis, and five genes (*polL-P*) were assigned as the roles for CPOAA biosynthesis [9,10].

As proposed in previous studies, CPOAA is formed by first transfer of an N-acetyl group to L-glutamate by PolN to form N-acetyl-L-glutamate, followed by phosphorylation to form N-acetyl-L-glutamate phosphate by PolP before stepwise reduction, deacylation, transcarbamoylation and hydroxylation (Figure 1A) [9]. Remarkably, the role of PolO as carbamoyltransferase catalyzing α-amino-δ-hydroxyvaleric acid (AHV) to generate α-amino-δ-hydroxyvaleric carbamoylhydroxyvaleric acid (ACV) was unambiguously demonstrated by *in vivo* and *in vitro* experiments [9]. According to the proposed pathway, the first two steps were identical to those for the L-arginine biosynthetic pathway, in which the initial steps involve catalyzing L-glutamate to form N-acetyl-L-glutamyl-5-phosphate by sequential acylation and phosphoralation [9,11]. As an important amino acid, L-arginine plays key roles in many metabolic pathways, either primary or secondary [11]. The L-arginine biosynthetic pathway was distinctly characterized in bacteria including *E. coli* and *Streptomyces *[11]. In *E. coli*, there is a linear biosynthetic pathway for L-arginine biosynthesis [11], however, many other bacteria such as *Streptomyces* harbor cyclic L-arginine biosynthetic pathway [11-13], in which the N-acyl group of the intermediate N-acetyl-L-ornithine could be recycled as substrate for ArgA, therefore, the protein, ArgJ, in *Streptomyces* displays bifunctional roles for the acylation and deacylation [11].

Here we describe the genetic characterization of CPOAA biosynthesis with unveiling of the “pathway redundancy” in metabolic networks. *In silico* and genetic analysis of the polyoxin gene cluster demonstrated that five genes (*polL-P)* were involved in CPOAA biosynthesis; moreover, the inter-connections between metabolic pathways of polyoxin and L-arginine were systematically characterized by *in vivo* genetic experiments. All these will set the stage for the understanding of the “pathway redundancy” in metabolic networks, and pave the way for rational designing and optimizing the polyoxin biosynthetic pathway so as to increase antibiotic production *via* the strategies of pathway engineering.

## Results

### In silico analysis of the candidate genes for CPOAA biosynthesis

*In silico* analysis shows that the five genes (*polL-P*) in the *pol* cluster seem to be involved in biosynthesis of CPOAA. Among them, *polL* encodes a protein exhibiting no detectable homology to any proteins in the database, suggesting its obscure and unique catalytic mechanism for CPOAA biosynthesis; PolM is a 255-aa protein with considerable homology to short-chain dehydrogenase of *Pseudomonas aeruginosa* PAO1. Short-chain dehydrogenase is a very large family of enzymes, most of which are known to be NAD- or NADP-dependent oxidoreductases. The protein PolN shows low homology (32% identity) to amino-acid N-acetyltransferase of *Neisseria mucosa* ATCC 25996. PolP exhibits 54% identity to the acetylglutamate kinase of *Frankia alni* ACN14a, implying the similar catalytic mechanism of PolP in CPOAA biosynthesis. PolO displays significant homology to NodU, a carbamoyltransferase of *Sinorhizobium* sp [14], whose function was unambiguously confirmed as a carbamoyl transferase by previous *in vitro* experiments [9].

### Independent mutation of polL-N led to the accumulation of thymine polyoxin I

**Figure 2:**
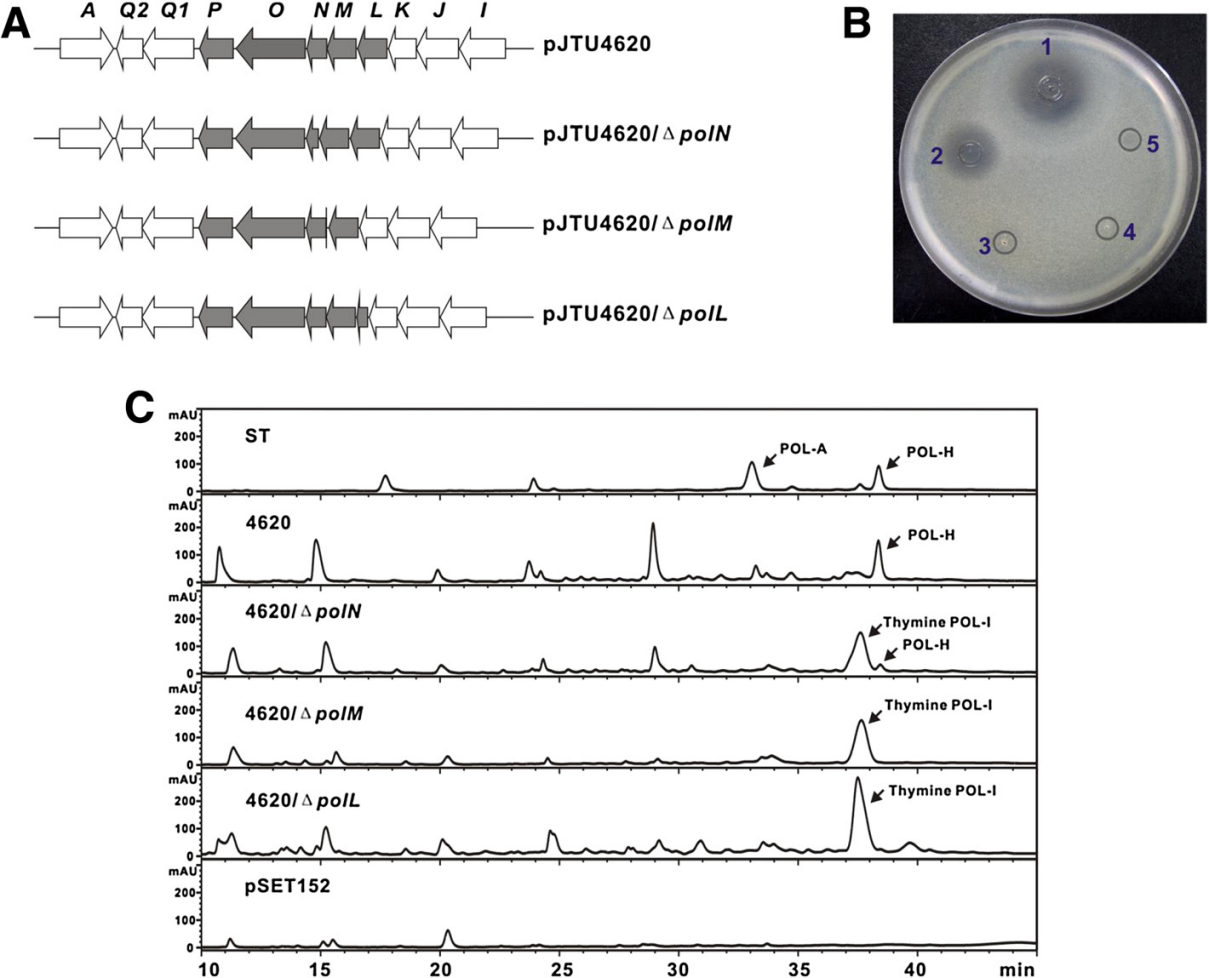
**Heterologous expression of the pJTU4620 derivatives (with individual mutation of *polN*, *polM *and *polL*) in *S. lividans *TK24. (A)** Schematic representation for the construction of pJTU4620 derivatives, the genes *polL-N* were independently in-frame-deleted in pJTU4620. **(B)** Bioassay for the related *S. lividans* TK24 recombinants, the sample of pJTU4620 (TK24 containing pJTU4620), pJTU4620/Δ*polN*, pJTU4620/Δ*polM* and pJTU4620*/*Δ*polL* were correspondingly spotted as 1-4; TK24 containing pSET152 (spot 5) was used as negative control. **(C)** HPLC analysis of the metabolites individually produced by the *S. lividans* TK24 recombinants. The authentic polyoxin standards (ST) and the sample of TK24 containing pJTU620 (4620) were used as positive controls, and TK24 containing pSET252 was used as negative control. The samples of TK24 bearing 4620 derivatives were individually indicated as 4620/Δ*polN*, pJTU4620/Δ*polM* and 4620/Δ*polL*. POL: polyoxin.

To demonstrate if *polL-N* play essential roles for CPOAA biosynthesis, all three genes were directly in-frame deleted in pJTU4620; after validation by PCR (Additional file 1: Figure S1), the resultant pJTU4620/*∆polL*, pJTU4620/*∆polM* and pJTU4620/*∆polN* cosmids (Figure 2A) were independently introduced into *S. lividans* TK24 for heterologous expression. Subsequently, the TK24 recombinants were fermented at 30°C for 3 d. After that, the broth was purified for bioassay and LC/MS analysis. Results shows that the broth of both TK24 recombinants individually containing pJTU4620/*∆polL* and pJTU4620/*∆polM* has lost bioactivity against the indicator fungi, *Trichosporon cutaneum* (Figure 2B)*,* while the TK24 recombinant bearing pJTU4620/*∆polN* was found to possess decreased bioactivity against *Trichosporon cutaneum* in comparison with the TK24 harboring intact pJTU4620 (Figure 2B)*.* Further HPLC analysis indicated the three TK24 recombinants obtained the ability to make a novel intermediate with abolishment/decreased polyoxin H production (Figure 2C). For the identification of the novel intermediate, it was subjected to MS and MS/MS analysis, and results showed that the intermediate generates [M+H]^+^ ion at *m/z =* 411.1 (Additional file 1: Figure S2), which was further fragmented into distinctive ions of *ca.* 285.0, 267.0, 183.0, and *etc.* (Additional file 1: Figure S2 and Figure S4), consistent with those generated by thymine polyoxin I authentic standard (Additional file 1: Figure S4); all the data suggesting that *polL-N* are responsible for CPOAA biosynthesis, simultaneously, implicating that a homologous gene is existed within the genome of *S. lividans* TK24 partially complementing *polN* mutant to restore polyoxin H production.

### polN possesses the ability to restore the growth phenotype for E. coli argA mutant

**Figure 3:**
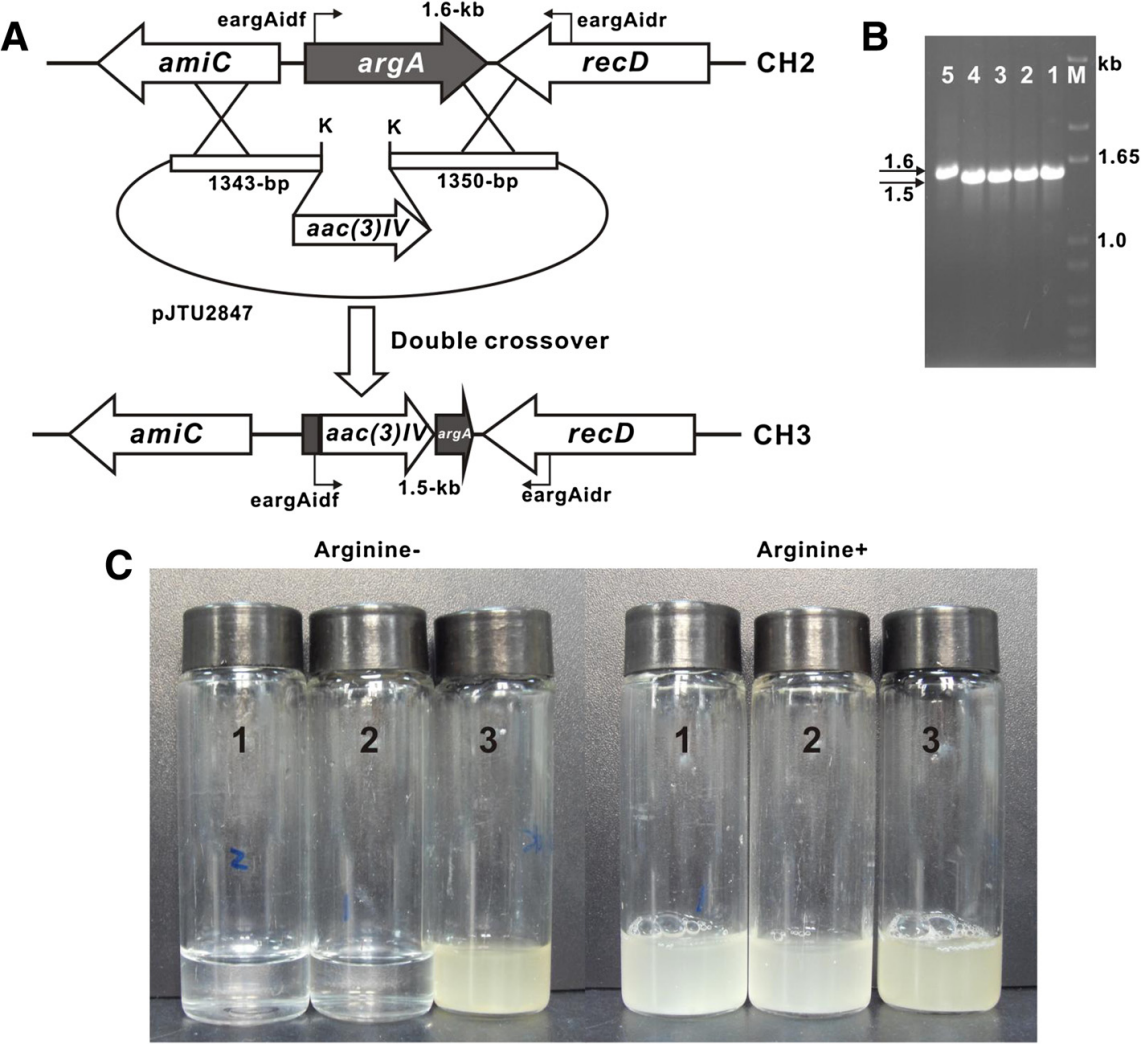
**Targeted inactivation of *argA *in *E. coli *and mutant complementation by *polN. *(A)** Representational map for construction of the CH3 mutant. **(B)** Identification of CH2 mutants. As 1.1-kb *argA* fragment was replaced by 1.0-kb *aac(3)IV* gene, the PCR product for CH3 mutants was ca. 1.5-kb, while the wild type produces 1.6-kb PCR product. **(C)** Complementation of CH3 mutant by *polN*. CH3 mutant of *E. coli* BL21(DE3) (1) and CH3 mutant containing pET28a (2) were used as negative controls, while CH3 mutant containing pJTU2838 with inserted *polN* was indicated as (3). The final concentration for arginine and thymidine used in this experiment is 50 μg/ml and 200 μg/ml, respectively, and the liquid cultures were incubated at 37°C for 90 h.

To get direct genetic evidence that *polN* functions as N-acetylglutamate synthase (ArgA), *E. coli thyA* mutant (CH2) was first constructed, validated by PCR (Additional file 1: Figure S3), and plate grown experiments showed that the *thyA* (thymidylate synthase gene) mutant abolishes growth phenotype without exogenous thymidine, demonstrating the correct *thyA-*phenotype of CH2 mutant (Additional file 1: Figure S3). CH3 mutant was further constructed on the basis of CH2 mutant using *thyA* as anti-selective marker [15]. PCR results showed that the CH2 mutant could produce 1.6-kb product, while that of the candidate CH3 mutants was 1.5-kb (Figure 3A, B). To investigate whether *polN* could complement CH3 mutant, pJTU2838 bearing *polN* was introduced into the mutant CH3, and complementation experiments indicated that *polN* harbors the ability to restore the growth phenotype for CH3 mutant, moreover, when grown in M9 liquid medium for 90 h, the OD_600_ of the complemented strain is 2.065, while the negative controls (CH3 and CH3/pET28a) almost stand still compared with the initial growth status (Figure 3C, Additional file 1: Table S3); all these strongly suggesting that *polN* is capable of cross-complementing with *argA* in L-arginine biosynthetic pathway.

### Targeted disruption of polP partially affects polyoxin production

**Figure 4:**
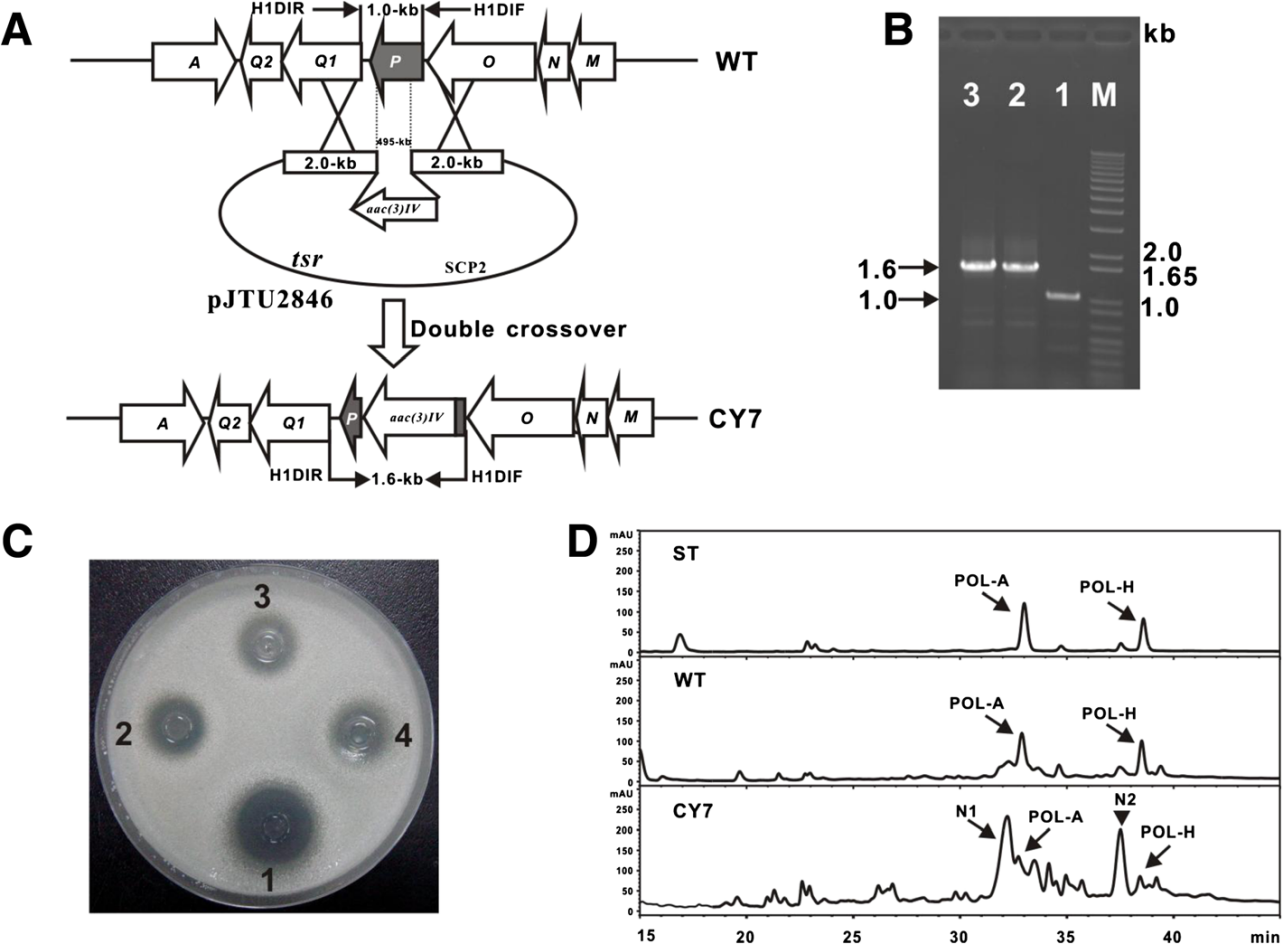
**Targeted inactivation of *polP. *(A)** Schematic representation for the construction of CY7 mutant. **(B)** PCR confirmation of the CY7 mutants, as a 1.1-kb *aac(3)IV* fragment replaced 495-bp of *polP*, the CY7 mutants give 1.6-kb PCR product, while WT of *S. cacaoi* produces 1.0-kb product. **(C)** Bioassay of the metabolites produced by CY7 mutant, the sample of *S. cacaoi* wild type indicated as (1) was used as positive control, and the samples from CY7 mutants were indicated as (2-4). 35 μl sample (supernatant) was used for all bioassays in this study. **(D)** HPLC analysis of the metabolites produced by CY7 mutant. Polyoxins authentic standard (ST) and sample from wild-type of *S. cacaoi* (WT) were used as positive control, and the sample from CY7 mutant was indicated as CY7.

To demonstrate the positive role of *polP* involved in CPOAA biosynthesis, the *polP* disruption vector, pJTU2846, was conjugated into *S. cacaoi*, after validation, the conjugants were released on MS medium for 7 d, subsequently, the spores were diluted with gradient on Apr^R^ MS medium, and random Apr^R^Thio^S^ candidate mutants were selected for further PCR confirmation. Results shows that the wild type of *S. cacaoi* generates 1.0-kb product, and the PCR product of the two candidate *polP* mutants (CY7) is 1.6-kb in size (Figure 4B), indicating that *polP* was successfully disrupted. For detection of resultant phenotype of CY7, the two mutant strains were cultivated for fermentation at 30°C for 3 d; after that, the broth was further analyzed by bioassay and HPLC. Compared with wild type of *S. cacaoi*, the CY7 samples shows apparently decreased bioactivity against the indicator fungi (Figure 4C), and HPLC analysis displayed that CY7 mutants partially lost the ability to produce polyoxin (bioactive components against the indicator fungi), meanwhile, the CY7 mutants also accumulate two novel intermediates designated as N1 and N2 (Figure 4C).

To further confirm the identities of the two intermediates (N1 and N2), both peaks were analyzed by LC/MS, and the results showed that the peaks at 33.1 min and 36.5 min could produce the [M+H]^+^ ions at *m/z* = 427.3 and 411.4, respectively, which corresponds to the authentic standards of polyoxin I and thymine polyoxin I (Additional file 1: Figure S4), moreover, MS/MS fragmentation pattern of the two peaks were in consistent with the standards (Additional file 1: Figure S4), suggesting that *polP* is not essential for CPOAA biosynthesis but only for its maximal production, likewise, indicating a homolog existing to rescue the *polP* mutation.

### polP is capable of complementing argB mutant of Streptomyces coelicolor

**Figure 5:**
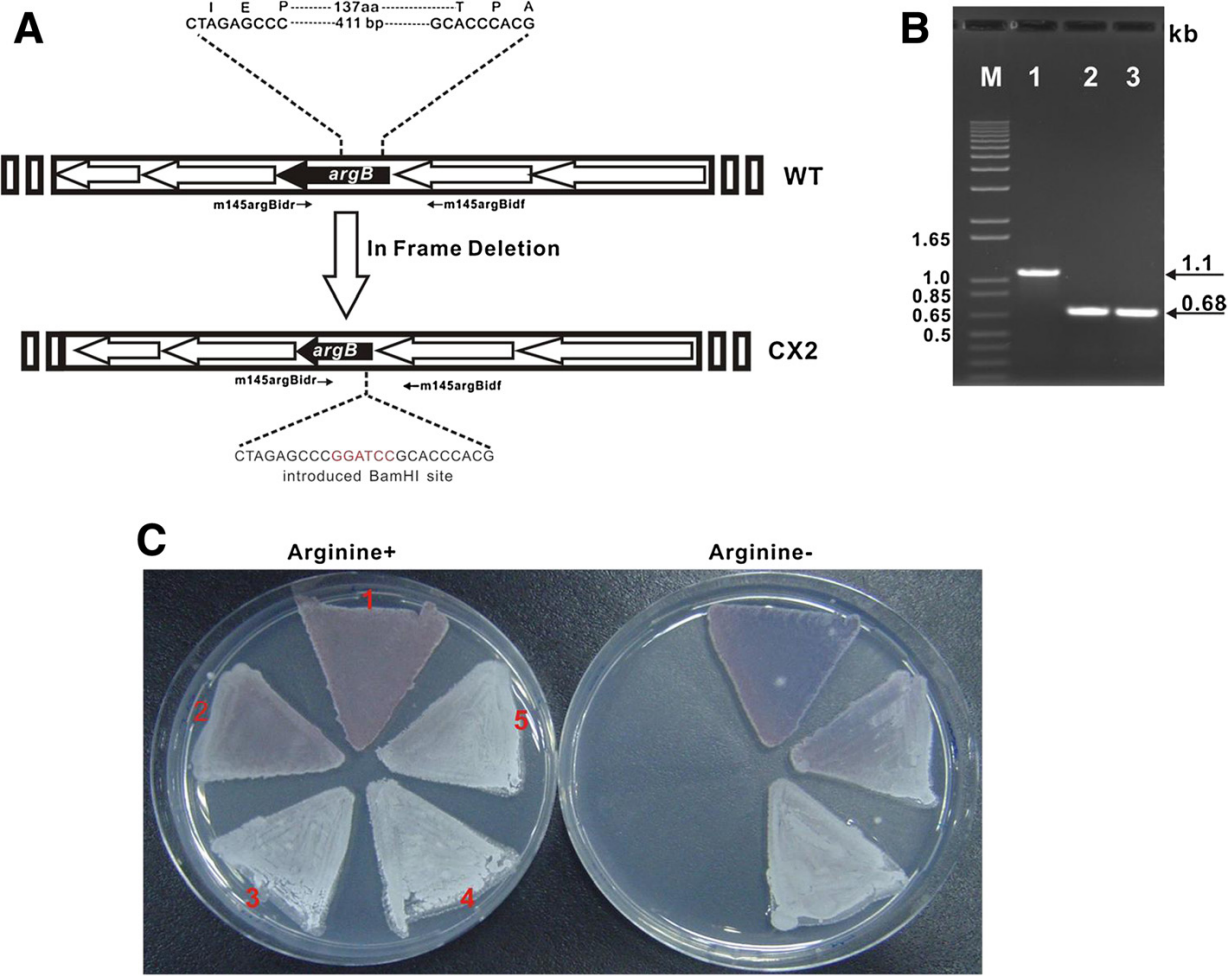
**In frame deletion of *argB *in *S. coelicolor *A3(2) and mutant complementation by *polP. *A.** Schematic representation for construction of CX2 mutant; **B.** PCR identification of CX2 mutant. As ca. 0.4-kb of *argB* was in frame deleted, the wild type of *S. coelicolor* A3(2) gives a 1.1-kb product, and the products of CX2 mutant are 0.68-kb in size. **C.** Plate grown experiments for CX2 mutant and its complemented strains, *S. coelicolor* A3(2) wild type (1) and CX2 mutant complemented by *argB* (*S. cacaoi*) in pJTU4713 (5) were used as positive controls, and CX2 mutant containing empty vector, pJTU2170, was used as negative control (3); CX2 mutant (2) and its complemented strain by *polP* in pJTU2870 (4) were indicated as parallels.

To deduce whether *polP* could cross-complement with its homolog, ArgB (N-acetyl glutamate kinase), the *argB* gene in *S. cacaoi* was cloned (Additional file 1, HQ202571) and sequenced as positive control for this study. Bioinformatic analysis showed that ArgB of *S. cacaoi* exhibit 95% identity to that of *Streptomyces pristinaespiralis*. For deep investigation of *in vivo* function of *polP*, the *argB* in frame deletion construct pJTU4710 was conjugated into *S. coelicolor,* then the *argB* mutant (CX2) was screened according to the standard protocols (Figure 5A) [16]. As confirmed by PCR, the CX2 mutants give 0.68-kb product, whereas that of the wild type of *S. coelicolor* is 1.1-kb in size (Figure 5B). To see if pJTU2870 (*polP*) could complement the CX2 mutant to restore growth phenotype; the vector was introduced into CX2 for complementation. As Figure 5C showed, *polP* could entirely complement *argB* mutant of *S. coelicolor* (CX2) (Figure 5C), while the negative controls (CX2 and CX2/pJTU2170) display the lethal phenotype without exogenous L-arginine. Additionally, pJTU2838 (*polP*) was used to complement *argB* mutant of *E. coli*, and results show that *polP* complemented strain harbors the similar growth status to that complemented by *argB* (*S. cacaoi*) (Additional file 1: Table S4 and Figure S5), all the data suggesting that *polP* harbors identical *in vivo* functions to *argB*, and further establishing that there is a robust “cross-complementation” between them.

### Natural argB plays essential roles for both polyoxin production and L-arginine biosynthesis in S. cacaoi

To investigate whether *argB* mutant of *S. cacaoi* could survive the growth environment without exogenous L-arginine, the mutant (CY21) was constructed (Additional file 1: Figure S6). In contrast with our expectations, the CY21 mutants were unable to grow without added L-arginine (Additional file 1: Figure S6). Furthermore, the growth phenotype was entirely complemented by an introduced *argB* (CY21/pJTU4713) (Additional file 1: Figure S6)*,* suggesting that the natural *polP* is not capable of complementing the CY21 mutant, simultaneously excluding the possibility of a polar effect (or frameshift mutation).

**Figure 6:**
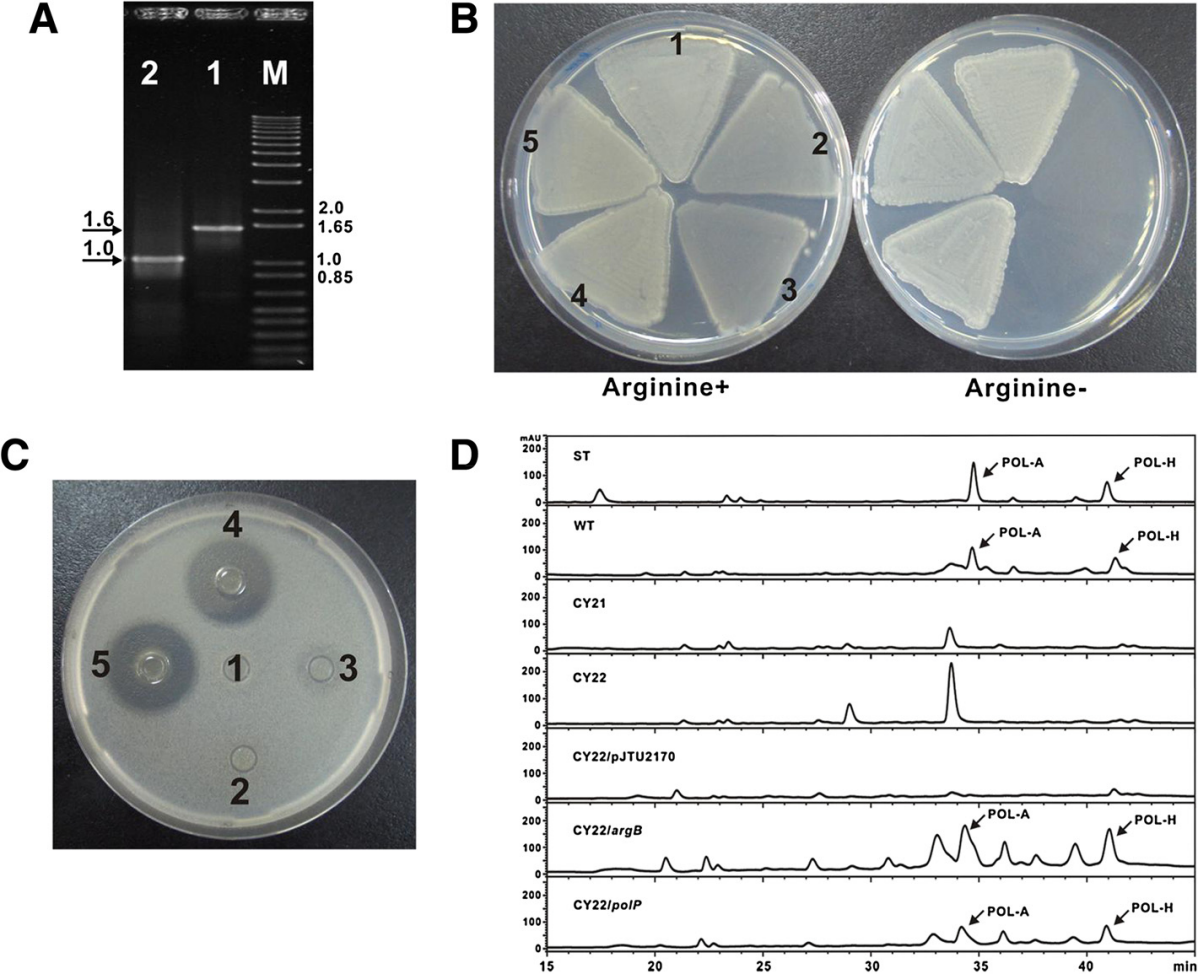
**Natural *argB *plays essential roles for both polyoxin production and L-arginine biosynthesis. (A)** PCR identification of CY22 mutant, the construction process is identical to that of CY7 mutant except that the CY21 mutant was used as start strain, and PCR product of CY22 mutant (1) was compared with that of CY21 mutant (2). **(B)** Growth phenotype of CY22 and its related complemented strains in minimal medium. CY7 mutant was used as positive control (1), and CY22 mutant (2) as well as its complemented strain (CY22/pJTU2170) with an empty vector (3) were selected as negative controls, CY22/pJTU4713 (*argB*) (4) and CY22/pJTU2870 (*polP*) (5) were detected as parallels. **(C)** Bioassay for the metabolites produced by CY22 and its related complemented strains. Samples of CY21 mutant (1), CY22 mutant (2), and CY22/pJTU2170 (3) were detected in parallel with those of CY22/pJTU4713 (*argB*) (4) and CY22/pJTU2870 (*polP*) (5). **(D)** HPLC analysis of the metabolites produced by the CY22 mutant and its related strains. Polyoxin authentic standard (ST) and metabolites produced by *S. cacaoi* wild type (WT) were used as positive controls, while the metabolites produced by CY21, CY22, CY22/pJTU2170 were utilized as negative controls; the samples of CY22/*argB* (pJTU4713) and CY22/*polP* (pJTU2870) were detected as parallels.

To further investigate into the interesting and unexpected phenotypes, *argB* and *polP* double mutant (CY22) was constructed using CY21 as starter strain according to the standard protocols [16], as PCR results showed that the CY21 mutant is able to give 1.0-kb product, and the product of CY22 mutant is 1.6-kb in size (Figure 6A). Plate growth experiments indicated that CY22 mutant has completely lost growth phenotype without exogenous L-arginine (Figure 6B), while *polP* (pJTU2870) and *argB* (pJTU4713) complemented strains were found to restore growth phenotype, and the negative control CY22/pJTU2170 displays lethal phenotype if no exogenous L-arginine added (Figure 6B), in full consistence with the expectations, thus suggesting that *polP* and *argB* share the same *in vivo* function to restore growth phenotype for the CY22 mutant.

To further see if the phenotype of CY7 was conferred by *argB*, the CY22 mutant and its complemented strains were inoculated for fermentations, after that, the preprocessed broth were subjected to bioassay analysis, and the results showed that the samples of CY22 and CY22/pJU2170 have abolished bioactivity against the indicator strain, *Trichosporon cutaneum* (Figure 6C). More interestingly and unexpectedly, the sample of the CY21 mutant has also been deprived of the bioactivity against the indicator strain, however, the CY22 mutant correspondingly complemented by *polP* and *argB* regained the bioactivity (Figure 6C). Further HPLC analysis indicated that the samples of CY21, CY22 and CY22/pJTU2170 were not capable of producing the distinctive peaks of either polyoxin A or polyoxin H as indicated by the standards at 34.8 min and 40.9 min, respectively, while the *polP* or *argB* complemented strains restored the abilities to produce the characteristic peaks at corresponding positions (Figure 6D).

**Figure 7:**
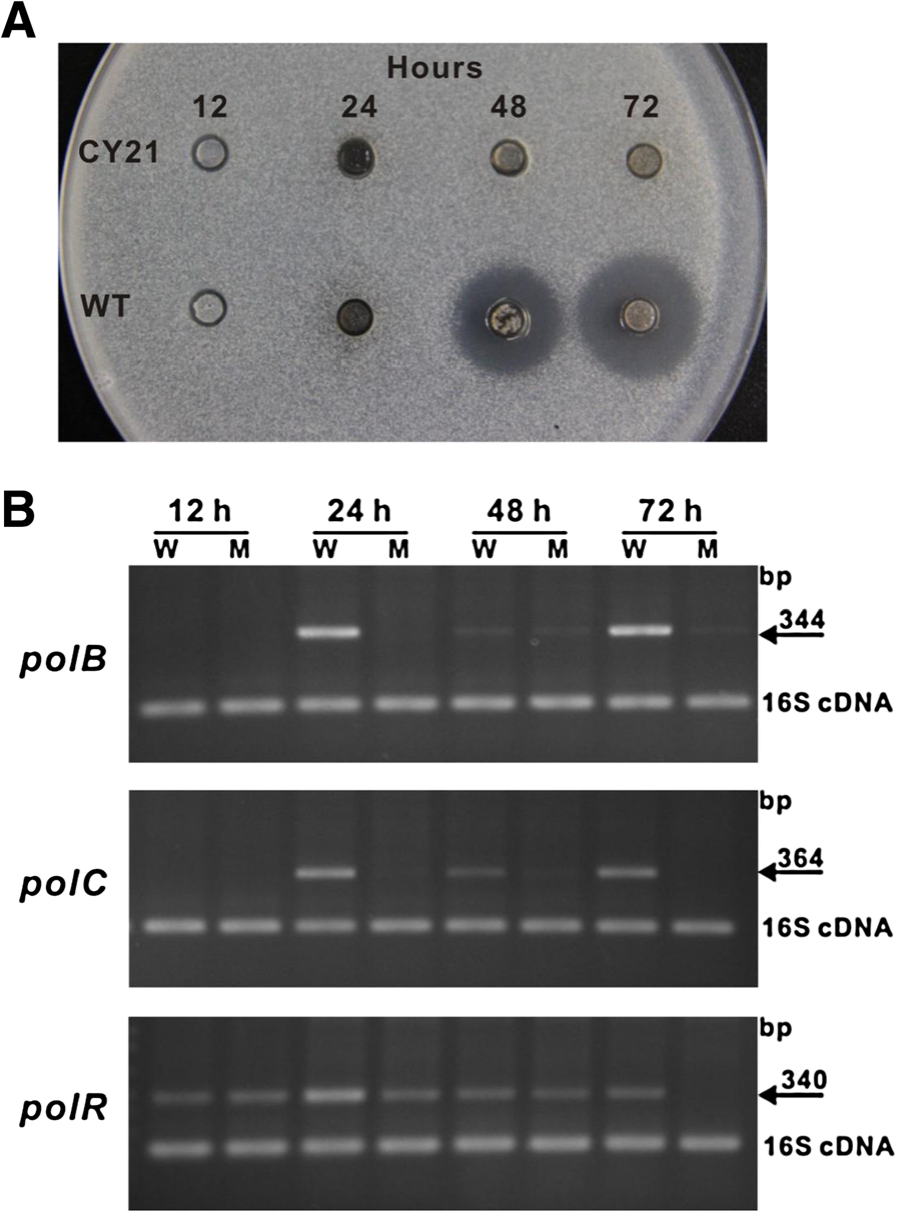
**Time-course bioassay and transcriptional analysis of *S. cacaoi *CY21 strain (*argB *mutant). (A)** Time-course bioassay for CY21 and WT strains of *S. cacaoi*; **(B)** Time-course transcriptional analysis of *S.cacaoi* CY21 strain, and RT-PCR was used for time-course transcriptional analysis of CY21 strain. “W” indicates wild type of *S. cacaoi,* and “M” means CY21 mutant of *S. cacaoi.* The methods for the time-course bioassay and time-course transcriptional analysis of *S. cacaoi* CY21 strain were described in Additional file 1.

For further accounting for the phenotype of CY21 mutant, the time-course transcriptional analysis and bioassay of the WT and CY21 strains of *S. cacaoi* were performed*,* and it is revealed that the transcription of the *pol* genes in CY21 was significantly affected (Figure 7A, B), which directly result in the abolishment of polyoxin production for CY21 strain, all these strongly suggesting that *polP* could cross-complement with *argB*, and revealing that *argB* plays essential role not only for L-arginine biosynthesis but also for polyoxin production.

## Discussion

Nucleoside antibiotics are a family of secondary metabolites whose biosynthetic precursors are originated from primary metabolisms including nucleotide (nucleoside), amino acids and saccharides [1,2,17]. Previous experiments assigned the biosynthetic precursors for the three building blocks (nucleoside skeleton, POIA and CPOAA) of polyoxin as UMP, L-isoleucine and L-glutamate, respectively [2,9,18-20]. As for CPOAA biosynthesis, the former labeling results led to a proposal that the biosynthesis was initiated by catalyzing L-glutamate to produce L-glutamate-γ-semialdehyde, which was finally converted to CPOAA with stepwise reactions [19,21].

The polyoxin biosynthetic gene cluster was previously cloned and sequenced [9], and further bioinformatic insights into the polyoxin biosynthetic gene cluster contradict to the previously-deduced pathway. For one thing, the hypothetic L-glutamate-γ-semialdehyde in CPOAA biosynthetic pathway was also a confirmed intermediate for L-proline biosynthesis, and its automatic self-cyclization would significantly decrease the efficiency for CPOAA biosynthesis, moreover, the roles of PolN and PolP could not be appropriately assigned in the CPOAA biosynthetic pathway [9]. On the other hand, PolN pertains to GCN5 super family acetyl transferase, which is capable of utilizing broadly flexible substrate as acetyl group acceptor [22]. PolP function is easier to be determined for its significant homology to ArgB, a well characterized protein in L-arginine biosynthetic pathway [11,13]. Accordingly, it was tentatively proposed that L-glutamate was catalyzed by PolN to give rise to N-acetylglutamate, which was then converted to *N-*acetylglutamate 5-phosphate in the charge of PolP.

The existence of the demonstrated “pathway redundancy” between the biosynthetic pathways of polyoxin and L-arginine promoted us to ponder the interesting phenomena raised in this study. Since the roles of *polN* and *polP* were overlapped with those of *argA* and *argB* in L-arginine biosynthesis, it seemed as if PolN and PolP would be redundant and dispensable for polyoxin biosynthesis. However, microbial cells, more specifically, the polyoxin producer, have chronically evolved, and their cell factories should be highly efficient and economical [23]. In this respect, the existence of *polN* and *polP* seemed to be reasonable and necessary.

For microbial cells, L-arginine could be synthesized *via* its biosynthetic pathway; nevertheless, the biosynthesis would be partly decreased once the cells could detect the existence of L-arginine [11,24]. Put it another way, the switch for L-arginine biosynthesis was not in full turn-on status for microbial cells with consistence of exogenous L-arginine, from the point of view, *polN* and *polP* were indispensible for polyoxin biosynthesis. Consequently, mutants of *polN* or *polP* were only harbor partial capability of polyoxin production. Another interesting and unexpected phenomenon is the *argB* mutation in *S. cacaoi* could not be complemented by natural *polP*, and further research reveals *argB* plays essential role for both biosynthesis of polyoxin and L-arginine, namely, *argB* mutant simultaneously abolishes the phenotypes of growth and polyoxin production, partly addressing the interesting phenotype of CY21. For CY21 mutant, Mutation of *argB* means the L-arginine biosynthetic pathway was interrupted, with the circumstance occurred, the microbial cells would generate chain stringent responses to turn off unessential secondary metabolisms, such as polyoxin biosynthesis. The unusual and unexpected phenotypes found in this study promote us to explore the precise molecular mechanisms for the impact of L-arginine biosynthesis on regulation of polyoxin production, and the related research is now in progress.

“Pathway redundancy” in metabolic networks was widely distributed in nature, as the representative cases characterized in the biosynthetic pathways of FR008/candicidin [25] and clavulanic acid [26]. Indeed, the interconnection between different pathways including those of polyoxin and L-arginine suggests that the related proteins should be predominantly responsible for their individual business in the metabolic networks; as a result, “Cross-complementation” should be merely a part-time job.

## Conclusion

Five genes (*polL-P*) in the current research were identified to be involved in CPOAA biosynthesis, and *argA* and *argB* in L-arginine biosynthetic pathway contributed to the “cross-complementation” with CPOAA pathway, most interestingly, we found that *argB* in *S. cacaoi* is indispensible for both polyoxin production and L-arginine biosynthesis. These data should provide a case for the research on the “pathway redundancy” in metabolic networks, and lay a solid foundation for target improvement of polyoxin production *via* synthetic biology strategy.

## Materials and Methods

### Bacterial strains and plasmids (cosmids)

Bacterial strains and plasmids (cosmids) used in this research are described in Additional file 1: Table S1.

### General methods and culture conditions

General approaches for the manipulation of *E. coli* and *Streptomyces* were based on the standard methods of Sambrook *et al. *[27] or Kieser *et al. *[16]. *Strepmyces* were grown on MS agar or in TSB (YEME) liquid medium at 30°C [16]. Liquid fermentation medium (per liter containing: Soy powder 20 g, corn powder 20 g, soluble starch 20 g, glucose 10 g, Yeast extract 10 g, CaCO_3_ 4 g, K_2_HPO_4_ 2 g, NaNO_3_ 2 g, add tap water till 1 liter) was used for polyoxin production. The MM agar [16] and M9 medium [25] were used for detection of growth phenotype for the *arg* mutant of *E. coli* or *Streptomyces*. The final antibiotic concentration used in this study is as follows: ampicillin 100 μg/ml, apramycin 30 μg/ml, kanamycin 50 μg/ml, chloramphenicol 25 μg/ml and thiostrepton 12.5 μg/ml.

### DNA sequencing and sequence analysis

DNA sequencing was accomplished at Shanghai Maojor Ltd using Applied Biosystems Model 3730 automated DNA sequencer. Sequence data analysis was performed with the FramePlot online program (http://watson.nih.go.jp/~jun/cgi-bin/frameplot-3.0b.pl) [28]. Sequence homology searches were performed using the NCBI online BLAST software [29].

### Construction of pJTU4620 for mutational analysis of the target pol genes

For construction of pJTU4620, m5A7 cosmid [9] (containing XbaI and SpeI sites correspondingly located at the both sides of foreign insertion) was initially digested by XbaI, and the resultant cohesive ends were blunted by Klenow Fragment (Fermentas). After that, the blunted linear fragment was self-ligated to render pJTU4619, in which SpeI site was subsequently blocked to generate pJTU4620 with the method described as above.

### Independent mutation of polL-polN in pJTU4620 cosmid

For individual mutation of *polL-polN* in pJTU4620, PCR-targeting technology was performed, and the *neo* cassettes amplified by different pairs of the primers as described in Additional file 1: Table S2 were independently recombined into pJTU4620 to result in mutation of the target genes, subsequently, the *neo* cassette was removed from pJTU4620 by XbaI-SpeI double digestion leaving the in frame deletion scar.

### Construction and complementation of E.coli argA mutant (thyA and argA double mutant)

For construction of the *argA* mutant of *E. coli*, a 3.0-kb fragment containing *thyA* of *E. coli* was cloned into pKD46 [30] as negative selective marker, and CH2 (*thyA*^*-*^) mutant (Additional file 1: Figure S2) was used as starter strain, with primers eargAF1 & eargAR1 and eargAF2 & eargAR2-2, the double arms for *argA* disruption were independently cloned into pIJ2925 to give pJTU2835, then the EcoRI engineered double arms was cloned into pJTU2183 to give pJTU2836, finally, the *aac(3)IV* from pJTU2848 was inserted into the KpnI site of pJTU2836 to generate the *argA* disruption vector, pJTU2847. For the complementation of *argA,* an EcoRI-NdeI *polN* fragment from pJTU2930 was cloned into identical sites of pET28a to produce pJTU2838.

### Construction and identification of CY7 mutant

For construction of the CY7 mutant, two *polP* disruption arms were independently amplified with primers (H1L-armF with H1L-armR and H1R-armF with H1R-armR) and cloned in pBlueScriptII SK(+) to form pJTU2814 and pJTU2815, respectively; and then a XbaI-PstI engineered fragment from pJTU2814 was cloned into counterpart sites of pJTU2815 to produce pJTU2816, and the PstI engineered *aac(3)IV* fragment from pJTU2844 was cloned into pJTU2816 to result in pJTU2845, from which the XbaI-EcoRI engineered fragment was cloned into pHL212 (Tao *et al.*, unpublished) to give the *polP* disruption vector, pJTU2846. For identification of CY7 mutant, primers H1DIR and H1DIF were used.

### Construction, identification and complementation of CY21 and CY22 mutants

For construction of the CY21 mutant, a BglII engineered PCR fragment (primers caargBRf2 and caargBRR) was cloned into BglII-HpaI sites of pOJ446 to generate pJTU4730, and a BglII-XbaI PCR fragment (primers caargB1f and caargBLR2) was inserted into corresponding sites of pJTU4730 to result in pJTU4731. After that, a BglII fragment bearing *tsr* was cloned into counterpart site of pJTU4731 to form pJTU4731-*tsr*. PCR with primers caargBef and aegDR was used to identify CY21 mutants. For construction CY22 mutant, *polP* was further disrupted using CY21 as start strain according to the method described as above. For the complementation of CY21 and CY22, *polP* and *argB* were individually cloned into pJTU2170 to form pJTU2870 and pJTU4713.

### Construction and complementation of the argB mutant of S. coelicolor A3(2) (CX2)

For construction of the CX2 mutant, the left arm for *argB* mutation was amplified by KOD-plus (Toyobo) polymerase with primers M145argBLF and M145argBLR, after treated by XbaI, the fragment was cloned into XbaI-EcoRV sites of pOJ260 to give pJTU4709, then the EcoRI-BamHI right arm PCR product amplified with primer M145argBRF M145argBRR was cloned into corresponding sites of pJTU4709 to generate the *argB* in frame deletion vector, pJTU4710. After that, this vector was conjugated into *S. coelicolor* A3(2) for construction of *argB* in frame deletion mutant (CX2) based on the standard protocols [16]. For complementation of *argB* mutant of *S. coelicolor*, NdeI-EcoRI fragments containing *polP* (pJTU2829) and *argB* (pJTU2883) was individually inserted into pJTU2170 to generate pJTU2870 and pJTU4713.

### Purification and assay of polyoxin

Polyoxin produced by *S. lividans* TK24, *S. cacaoi* as well as its derivatives was detected by bioassay and LC/MS with Agilent 1100 series LC/MSD Trap system. For the bioassay, *Trichosporon cutaneum* was used as indicator strain, and the protocol were according to Chen *et al. *[9]. For the purification and HPLC analysis of polyoxin, the methods were based on Chen *at al *[9], and the targeted fraction was collected and condensed before LC/MS analysis.

### Conditions for LC/MS analysis

The conditions performed for LC/MS analysis were as follows: Agilent ZORBAX SB-C_18_ column (4.6 × 250 mm), flow rate 0.3 ml/min at room temperature with elution gradient 5%-40% Methanol: 0.3% TFA (HPLC grade) over 40 min at 0.3 ml/min [31]. The elution was monitored at 262 nm with a DAD detector and the data were analyzed with Agilent data analysis software.

### Accession number

The nucleotide and protein sequences reported in this paper have been deposited in GenBank under the accession number HQ202571.

## Competing interests

The authors declare that they have no competing interests.

## Authors’ contributions

WC, DD and CW carried out experiments, analyzed the primary data, WC wrote the draft manuscript. TH and LZ assisted with experiments. ZD supervised the whole research work and revised the manuscript. All authors read and approved the final manuscript.

## Supplementary Material

Additional file 1: Table S1 Strains, plasmids and cosmids used in this study. **Table S2**: PCR primers used in this study. **Table S3**: Growth status for CH3 mutant and its complemented strains. **Table S4**: Growth status for CH4 mutant and its complemented strains. **Figure S1**: Identification of pJTU4620 derivatives by PCR. (A) Identification of pJTU620/*∆polL*. (B) Identification of pJTU620/*∆polM*. (C) Identification of pJTU620/*∆polN*. **Figure S2**: MS analysis of the metabolites produced by pJTU4620 derivatives. **Figure S3**: Constrution of CH2 mutants and confirmation of its related biological phenotype. (A). Representational map for the construction of CH2 mutant; (B). Identification of CH2 mutants, M: 1 kb plus ladder, 1: *E. coli* BL21(DE3) wild type, 2-5: *E. coli* BL21(DE3) CH2 mutants; (C). Confirmation of the biological phenotype of CH2 mutants, 1: *E. coli* BL21(DE3) Wild type; 2-5: *E. coli* BL21(DE3) CH2 mutants. **Figure S4**: LC/MS analysis of the metabolites produced by CY7 mutant. ST/(Thymine) POL-I: (Thymine) POL-I authentic standard, ST/N1(N2): Novel compounds N1(N2) produced by CY7 mutant. **Figure S5**: Construction and Complementation of the CH4 Mutant. (A). Representational map for construction of CH4 mutants; (B). PCR identification of CH4 mutants, M: 1 kb plus ladder, 1: *E .coli* BL21(DE3) wild type, 2-3: CH4 mutants; (C). Minimal broth grown experiments for CH4 mutant and its complemented strains, 1: CH4 mutant, 2: CH4/pET28a, 3: CH4/pJTU2837, 4: CH4/pJTU2884, 5: *E. coli* BL21(DE3). **Figure S6**: Costruction and complementation of CY21 mutant. (A). Representational map for construction of CY21 mutants; (B). PCR identification of CY21, M: 1 kb plus ladder, 1: *S. cacaoi* WT, 2-4: f *S. cacaoi* CY21 mutants; (C). Plate grown experiments for CY21 mutant and its complemented strain, 1: CY21 mutant, 2: CY21 mutant containing pJTU2170 as negative control, 3: CY21 mutant containing pJTU4713 (*argB* gene inserted into pJTU2170), 4: *S. cacaoi* wild type. Click here for file
