# Supplementary material for: Genetic dissection of the polyoxin building block-carbamoylpolyoxamic acid biosynthesis revealing the “pathway redundancy” in metabolic networks
Source: Microb Cell Fact. 2013 Dec 7;12:121. doi: 10.1186/1475-2859-12-121 (PMC4029187; doi:10.1186/1475-2859-12-121)
Supplement: Additional file 1: Table S1 — Strains, plasmids and cosmids used in this study. Table S2: PCR primers used in this study. Table S3: Growth status for CH3 mutant and its complemented strains. Table S4: Growth status for CH4 mutant and its complemented strains. Figure S1: Identification of pJTU4620 derivatives by PCR. (A) Identification of pJTU620/∆polL. (B) Identification of pJTU620/∆polM. (C) Identification of pJTU620/∆polN. Figure S2: MS analysis of the metabolites produced by pJTU4620 derivatives. Figure S3: Constrution of CH2 mutants and confirmation of its related biological phenotype. (A). Representational map for the construction of CH2 mutant; (B). Identification of CH2 mutants, M: 1 kb plus ladder, 1: E. coli BL21(DE3) wild type, 2-5: E. coli BL21(DE3) CH2 mutants; (C). Confirmation of the biological phenotype of CH2 mutants, 1: E. coli BL21(DE3) Wild type; 2-5: E. coli BL21(DE3) CH2 mutants. Figure S4: LC/MS analysis of the metabolites produced by CY7 mutant. ST/(Thymine) POL-I: (Thymine) POL-I authentic standard, ST/N1(N2): Novel compounds N1(N2) produced by CY7 mutant. Figure S5: Construction and Complementation of the CH4 Mutant. (A). Representational map for construction of CH4 mutants; (B). PCR identification of CH4 mutants, M: 1 kb plus ladder, 1: E .coli BL21(DE3) wild type, 2-3: CH4 mutants; (C). Minimal broth grown experiments for CH4 mutant and its complemented strains, 1: CH4 mutant, 2: CH4/pET28a, 3: CH4/pJTU2837, 4: CH4/pJTU2884, 5: E. coli BL21(DE3). Figure S6: Costruction and complementation of CY21 mutant. (A). Representational map for construction of CY21 mutants; (B). PCR identification of CY21, M: 1 kb plus ladder, 1: S. cacaoi WT, 2-4: f S. cacaoi CY21 mutants; (C). Plate grown experiments for CY21 mutant and its complemented strain, 1: CY21 mutant, 2: CY21 mutant containing pJTU2170 as negative control, 3: CY21 mutant containing pJTU4713 (argB gene inserted into pJTU2170), 4: S. cacaoi wild type. [file 1475-2859-12-121-S1.doc]

**Supplemental Data**

**Table S1. Strains, plasmids and cosmids used in this study**

| **Strain**/  **Plasmid** /  **Cosmid** | **Relevant characteristics*** | **Reference or source** |
| --- | --- | --- |
| ***S. cacaoi* strains** |  |  |
| WT | Wild-type strain producing polyoxin |  |
| CY7 | *polP* mutant with insertion of *aac(3)IV* | This study |
| CY21 | *argB* mutant of *S.cacaoi* | This study |
| CY22 | *polP* and *argB* double mutant | This study |
| *S. coelicolor* A3(2) | Model strain of *Streptomyces* |  |
| *S. coelicolor* CX2 | *argB* mutant of *S. coelicolor* A3(2) | This study |
| *S. lividans* TK24 | Model strain of *Streptomyces* | This study |
| ***E. coli* strains** |  |  |
| DH10B | F^-^ *mcr*A Δ(*mrr*-*hsd*RMS-*mcr*BC) φ80d *lac*ZΔM15 Δ*lac*X74 *deo*R *rec*A1*end*A1*ara*Δ139 D(*ara*, *leu*)7697 *gal*U *gal*K λ^-^ *rps*L *nup*G | GIBCO BRL |
| ET12567 (pUZ8002) | *dam dcm hsd*S pUZ8002 |  |
| BL21(DE3)pLysE | F –, *ompT, hsdS_B_ (r_B_^–^ m_B_^–^), gal ,dcm* (DE3),  pLysE (Cm^R^) | Stratagene |
| CH2 | *thyA* mutant of BL21(DE3)pLysE | This study |
| CH3 | *thyA* and *argA* double mutant of BL21(DE3)pLysE | This study |
| CH4 | *argB* mutant of BL21(DE3)pLysE | This study |
| ***Trichosporon cutaneum*** | Indicator fungi used for the bioassay of polyoxin | CGMCC |
| **Plasmids** |  |  |
| pIJ2925 | *bla, lac*Z |  |
| pBlueScriptII SK(+) | *bla, lac*Z, *ori*f1 (SK+, hereafter) | Stratagene |
| pMD18-T | pUC18 derived T-vector | TaKaRa |
| pSET152 | *aa (3)I V*, *lacZ*, rep^puc^, attФC31, oriT |  |
| pOJ446 | *aa (3)I V,* SCP2, rep^puc^, attФC31, oriT |  |
| pIB139 | A pSET152 derivative containing *ermE** promoter |  |
| pKD46 | *bla*, *ara, oriR101, rep101^ts^* |  |
| pIJ773 | A vector containing *aac(3)IV* used for PCR-targeting |  |
| pIJ790 | A helper plasmid containing *aac(3)IV* used for PCR-targeting |  |
| pHL212 | A vector used for gene disruption | (Tao *et al*.unpublished) |
| pOJ260 | A suicide plasmid for gene disruption in *Streptomyces* |  |
| pJTU2170 | pIB139 derivative with insertion of *bla*, and replacement of *aac(3)IV* by *neo* | This study |
| pJTU2183 | pKD46 derivative with insertion of a PCR fragment carrying *thyA* from *E. coli* | This study |
| pJTU2814 | SK+ derivative carrying PstI-XbaI engineered PCR fragment containing left arm of *polP* disruption vector | This study |
| pJTU2815 | SK+ derivative carrying EcoRI engineered PCR fragment containing right arm of *polP* disruption vector | This study |
| pJTU2816 | pJTU2815 derivative carrying the PstI-XbaI engineered fragment from pJTU2814 | This study |
| pJTU2829 | SK+ derivative carrying structure gene of *polP* | This study |
| pJTU2830 | SK+ derivative carrying structure gene of *polN* | This study |
| pJTU2834 | pIJ2925 derivative carrying EcoRI-KpnI fragment for *E. coli* *argA* mutation | This study |
| pJTU2835 | pJTU2834 derivative bearing XbaI-KpnI fragment for *E. coli* *argA* mutation | This study |
| pJTU2836 | pJTU2183 derivative carrying XbaI-EcoRI engineered fragment from pJTU2835 | This study |
| pJTU2838 | pET28a derivative bearing *polN* structure gene | This study |
| pJTU2844 | SK+ derivative carrying EcoRI engineered PCR product containing *aac(3)IV* from pIJ773 | This study |
| pJTU2845 | pJTU2816 derivative bearing *aac(3)IV* from pJTU2844 | This study |
| pJTU2846 | pHL212 derivative containing XbaI-EcoRI engineered fragment *polP* disruption construct from pJTU2845 | This study |
| pJTU2847 | pJTU2836 derivative bearing *aac(3)IV* from pJTU2848 | This study |
| pJTU2848 | pMD18-T derivative carrying KpnI engineered fragment containing *aac(3)IV* from pIJ790 | This study |
| pJTU2865 | pIJ2925 derivative carrying 5.2-kb BglII fragment from  18F2 cosmid | This study |
| pJTU2870 | pJTU2170 derivative carrying NdeI-EcoRI engineered fragment containing *polP* from pJTU2829 | This study |
| pJTU2839 | pMD18-T carrying 0.3-kb PCR fragment containing partial *argB* of *S.cacaoi* | This study |
| pJTU2873 | pIJ2925 derivative containing 4.0-kb PvuII fragment bearing complete *argD* and partial *argB* from 9A6 cosmid | This study |
| pJTU2883 | SK+ derivative carrying *argB* amplified by PCR from 18F2 | This study |
| pJTU4701 | SK+ derivative bearing left arm for in frame deletion of *E. coli* *argB* | This study |
| pJTU4703 | SK+ derivative bearing right arm for in frame deletion of *E. coli* *argB* | This study |
| pJTU4704 | pKOV-kan derivative carrying *E. coli* disruption construct from pJTU4703 | This study |
| pJTU4709 | pOJ260 derivative carrying XbaI engineered left arm for in frame deletion of *S. coelicolor* *argB* | This study |
| pJTU4710 | pJTU4709 derivative carrying BamHI-EcoRI engineered right arm for in frame deletion of *S. coelicolor* *argB* | This study |
| pJTU4713 | pJTU2170 derivative carrying *argB* from pJTU2883 | This study |
| pJTU4730 | pOJ446 derivative with insertion of a BglII PCR fragment for *argB* mutation | This study |
| pJTU4731 | pJTU4730 derivative carrying BglII-XbaI right arm for *argB* mutation | This study |
| pJTU4731-*tsr* | pJTU4731 derivative bearing BglII engineered *tsr* fragment from pJTU2180 | This study |
| **Cosmid** |  | This study |
| m5A7 | A positive cosmid harboring complete gene cluster of polyoxin | This study |
| pJTU4620 | m5A7 derivative with XbaI and SpeI blocked | This study |
| 18F2 | A positive cosmid containing arginine biosynthetic genes | This study |
| 9A6 | A positive cosmid containing arginine biosynthetic genes | This study |

* *ori*T, origin of transfer of plasmid RK2; *tsr*, thiostrepton resistance gene; *aac(3)IV,* apramycin resistance gene; Cm^R^, chloramphenicol resistance gene; *neo*, Neomycin resistance gene; Kan, kanamycin resistance gene; CGMCC, China General Microbiological Culture Collection Center.

**Table S2. PCR primers used in this study**

| Name | Sequence （5’-3’） |
| --- | --- |
| eargAF1  eargAR1  eargAF2  eargAR2-2 | ggaattcTTACCAAACTTCAGGCTGTCGG  ggggtACCGCCGAGCATGATGACAA  ggggtACCACGCGCAGTATTCACTGGTT-3'  gctctagagaattCGCTGACCGATGAACAAAAGAA |
| H1L-armF:  H1L-armR | GCTCTAGA GCCAGGTCTCGGTGTTGTCG  AACTGCAG GTCCCGGTCGTCTCCAGCAT |
| H1R-armF:  H1R-armR: | AACTGCAG GACGGAGCCGCCGCACTTGA  GGAATTC ACCGGATCGGCGACTACCTGAC |
| thyAMF  thyAMR | ccaacccgcagtggcaatc  gcagtatggagcgaggaga |
| thyAIFDF  thyAIFDR | cgGGATCCTGTGACGTCTTCCTC  cgGGATCCGGTTCCGGTACGGTC |
| H1DF-f  H1DF-r  H3DF-f  H3DF-r | GTGGCGTCCAAGGGGTCGGT  GCGAGGGCGTCCTCTACCAG  GTTTCTCCATCTCCACGCTCAG  CATCGTCAACATCGGCTCCAT |
| eargAF1  eargAR1  eargAF2  eargAR2-2 | ggaattcTTACCAAACTTCAGGCTGTCGG  ggggtACCGCCGAGCATGATGACAA  ggggtACCACGCGCAGTATTCACTGGTT  gctctagagaatt CGCTGACCGATGAACAAAAGAA |
| argBF  argB2R | GCATCGTCAGCGAGTTCAAG  CGATCGAGGAGACGACCGG |
| KanB-F  KanB-R | cgggatcCAGCTATTCCAGAAGTAGT  cgggatcCTGGATGCCGACGGATTTG |
| aprelF  aprelR | ggaattctgCAGCGGAAAATGCAGCTCA  gctgcagCGGAATAGGAACTTCATGA |
| polJexF  polJexR | ccataTGACCACCGGAGCCCGCC  ggaattcTCAATCAGCGTCATGTCGTT |
| argDgood | GAGCCGATCCAGGGCGAGA  GCGGCTCGGTGAGCACGATA |
| k12argB1F k12argB1R | CCGTGGCGCTTATTGAAGG  ggaattcATTCACCAGTGCGCTAAA |
| k12argB2F k12argB2R | ggaattcAAAGCAGAACAACTGATT  CCACCAGATAATCCGCCAGTT |
| argDF  argDR | GAGCCGATCCAGGGCGAGA  GCGGCTCGGTGAGCACGATA |
| argD2F  argD2R | gagccgatccaaggcgaact  Cccgctccccggacataa |
| M145argBLF  M145argBLR | gctctagaCTCCTCCTCGGCGGTGAAGT  cgggatccCGTGGGTGCGTTGCTCGTT |
| M145argBRF  M145argBRR | cgggatccCCCGAGATCGACGGTGAA  ggaattcTGCTGTCCGTGACGGTGGTG |
| PolLtgtF | GTACCCGCCGCCCTCCAGGACAGCCTCAAGACCCTCGCG  TCTAGAGCTATTCCAGAAGT |
| polLtgtR | GTCGGTGAAGACCTTCTCGGTGATCAGCTCGTGCTCCGG  ACTAGTCTGGATGCCGACG |
| polL idF  polLidR | GCAATTCCATATGCTCACCCGACCCACG  GGAATTCTCACATGGGGTCGTAGCTC |
| polMtgtF | CAGGGCACGCACAGATGACGATTGCATGAGGTGGGGCAC  TCTAGAGCTATTCCAGAAGT |
| polMtgtR | GGTGCGTCCGTGGCCAGCGCCGGGCGTACGACGACGTCC  ACTAGTCTGGATGCCGACG |
| polM idF  polM idR | CGGCGACGCAGAGGTTGTA  GGGCACGCACAGATGACGA |
| polNtgtF | CGGCTCGGGGACGACCTGCTGCTGTACAACCTCTGCGTC  TCTAGAGCTATTCCAGAAGT |
| polNtgtR | CCATACGGCGGGCATGTGCTGGGGTTCGGTGCGGGTGAA  ACTAGTCTGGATGCCGACG |
| polNidf  polNidR | GTTTCTCCATCTCCACGCTCAG  CATCGTCAACATCGGCTCCAT |
| caargBRf2  caargBR2 | gaagatctTTCACCGACGAAGGCATC  gaagatctTTCACCGACGAAGGCATC |
| caargB1f  caargBLR2 | gctctagaGCCCGCATGGATTGCATAA  gaagatctGTCGATCATGGCGTTGCC |
| polBRTF  PolBRTR | AGCGATCTCGCCGTCGTCA  TGCTGGTGGTCGTCGGTGCT |
| polCRTF  polCRTR | TCCTTCCGCACCTGGCTGTC  AGCTCCTTCTTTCGGGCATC |
| polRRTF  polRRTR | AGCGGGTGCTGAGCATGTCA  AGAGCGAGGGTCCGGTGGTT |
| polYRTF  polYRTR | CGCCTTCCACGACCTGCTGA  GCTGTCTGGTCCTGCCATCTGC |

**Table S3. Growth status for CH3 mutant and its complemented st**rains

| Strain  Time | CH3  OD_600_ | CH3/pET28a  OD_600_ | CH3/ *polN*  OD_600_ |
| --- | --- | --- | --- |
| 0 h | 0.024 | 0.025 | 0.038 |
| 90 h (A-) | 0.010 | 0.024 | 2.065 |
| 90 h (A+) | 1.739 | 1.872 | 2.141 |

“A-”indicates no arginine added, “A+” means arginine added

**Table S4. Growth status for CH4 mutant and its complemented strains**

| Time  Strain | 0 h (OD_600_) | 70 h (OD_600_) | |
| --- | --- | --- | --- |
|  |  | Arg- | Arg+ |
| CH4 | 0.022 | 0.027 | 1.294 |
| CH4/pET28a | 0.024 | 0.026 | 1.249 |
| CH4/pJTU2838 | 0.024 | 1.334 | 2.002 |
| CH4/pJTU2884 | 0.009 | 1.740 | 1.607 |
| BL21(DE3) | 0.003 | 1.693 | 1.748 |

“A-” indicates no arginine added, and “A+” means arginine added

**Cloning and sequencing analysis of *argB* from *S. cacaoi* var. *asoensis.*** According to *argB* sequence of *S. coelicolor* A3(2) and *S. avermitilis,* a pair of primers argBF and argB2F was designed, and a distinct PCR product with expected 0.3-kb was amplified from the genome of *S. cacaoi*. Sequence analysis shows the polypeptide encoded by the fragment that the homology of the fragment is 95 % identity to ArgB of *S. avermitilis*. Using the primers, several positive cosmids were identified, and a 5.2-kb fragment from 18F2 and 4.0-kb BglII fragment from 9A6 was sequenced, and related sequence information was deposited in GenBank under accession no. number HQ202571.

**Construction and complemtation of *E. coli* CH4 mutant.** With primers k12argB1F with k12argB1R and k12argB2F with k12argB2R, double arms were amplified and independently cloned in to SK+ to pJTU4703, after that, a SalI-BamHI fragment was cloned into counterpart sites of pKOV-kan to produce the *argB* in frame deletion vector, pJTU4704. According to the method of Lalioti *et al* , the CH4 mutant was constructed. For complementation of CH4, pJTU2884 (*argB* of *S. cacaoi*) and pJTU2837 (*polP*) were constructed and transformed, and the resultant transformants were cultivated on liquid minimal medium to see the growth phenotype.

**Time course bioassay and time course Transcriptional analysis of the CY21 and WT strain of *S. cacaoi*.** The wide-type stain and CH21 were both grown in fermentation media at 30℃ with shaking at 220 r/m. The cells of the two strains, grown at 12 h, 24 h, 48 h and 72 h, were harvested by centrifugation, The cells were used for RNA extraction, and the supernatants (35 μl) were used for bioassay . The total RNA was extracted with the SBS total RNA isolation kit (Shanghai SBS Gene-tech Co., Ltd.), and quantified with NANODROP 2000 spectrophotometer (Thermo scientific). The digestion of DNA in the total RNA was performed in 50 μl of a reaction mixture containing 25 μg of total RNA, 5 μl of 10 ×DNaseI buffer with MgCl_2_ (Fermentas), 5μl of DNaseI(1 U/μl; Fermentas), 1 μl of RNase inhibitor (20 U/μl; Fermentas), and DEPC-treated water. The reaction mixture was incubated at 37℃ in a water bath for 1h. After incubation, 5 μl of EDTA(25 mM) was added to inactivate DNaseI, followed by incubation at 65℃ in a water bath for 10 min. PCR amplifying the 16s cDNA was performed to check the complete digestion of DNA. The 50 μl of PCR mixture contained 2 μl of inactivated DNaseI digestion mixture, 5 μl of 10 ×PCR buffer(rTaq buffer; TaKaRa), 5 μl of dNTP mixture(2.5 mM;TaKaRa), 1 μl each of primers 16sF and 16sR(10uM), 1 μl of 5 U/μl Taq DNA polymerase (rTaq; TaKaRa), and distilled water. The conditions for thermal cycling were denaturation at 94°C for 3 min followed by 30 cycles of denaturation at 94°C for 30 s, annealing at 60°C for 30 s, and extension at 72°C for 30 s. the sequences of the primers 16sF and 16sR are as follows: (16sF) 5’-AGTAACACGTGGGCAACTGC-3’/(16sR)5’-CTCAGACCAGTGTGGCCGGT-3’.The cDNA synthesis began with a reaction mixture containing 11 μl of inactivated DNaseI digestion mixture(5 ug total RNA), 1 μl of random hexamer primer (0.2 μg/μl; Fermentas). The mixture was incubated at 65°C for 5 min, and chilled on ice immediately. 4 μl of 5×reverse transcriptase buffer (RevertAid^TM^ H Minus reverse transcriptase buffer; Fermentas), 2 ul of dNTP (10 mM; Fermentas), 1 μl of RNase inhibitor (20 U/μl; Fermentas),1 μl of RevertAid^TM^ H Minus reverse transcriptase(200U/μl; Fermentas) were added. The mixture was incubated at 24°C for 10 min, 42°C for 1 h, and finally at 72°C for 10 min. PCR to amplify *polB*, *polC*, *polR*, *polY* and 16s rDNA respectively was performed with the synthesized cDNA using the primer pairs as follows: (*polB*-F)gcgc/(*polB*-R)gcgc; (*polC*-F)gcgc/(*polC*-R)gcgc; (*polR*-F)gcgc/(*polR*-R)gcgc; 16-sF/16sR.

**Supplemental Figures**

**
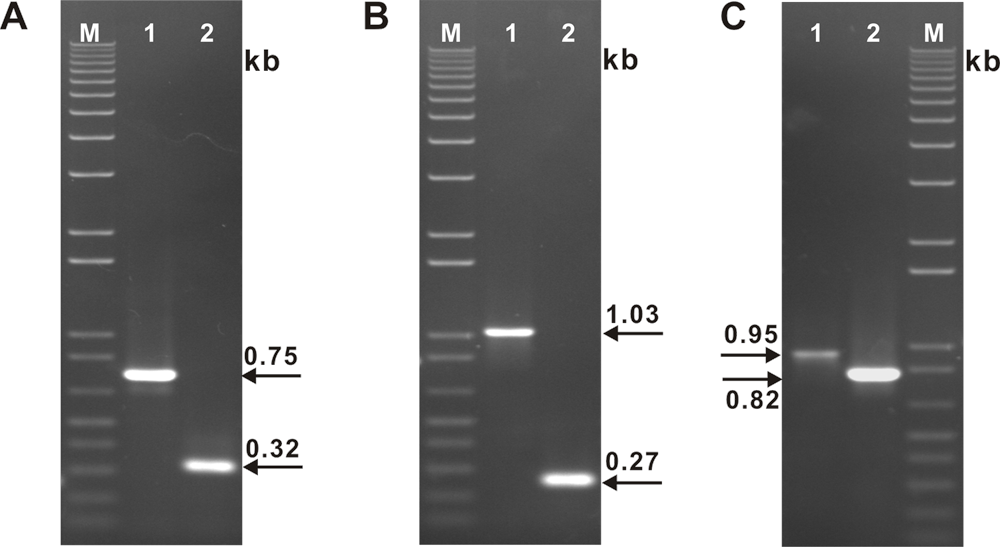
**

**Figure S1. Identification of pJTU4620 derivatives by PCR.** (A) Identification of *polL* mutation in pJTU620/*∆polL,* as 0.42-kb region was deleted from *polL*, the intact pJTU4620 gives 0.75-kb product, and pJTU620/*∆polL* 0.32-kb. (B) Identification of *polM* mutation in pJTU620/*∆polM,* as *ca.* 0.7-kb region was deleted from *polM*, the intact pJTU4620 gives 1.03-kb product, and pJTU620/*∆polM* 0.27-kb. (C) Identification of *polN* mutation in pJTU620/*∆polN,* as 0.13-kb region was deleted from *polN*, the intact pJTU4620 gives 0.95-kb product, and pJTU620/*∆polN* 0.82-kb.

**
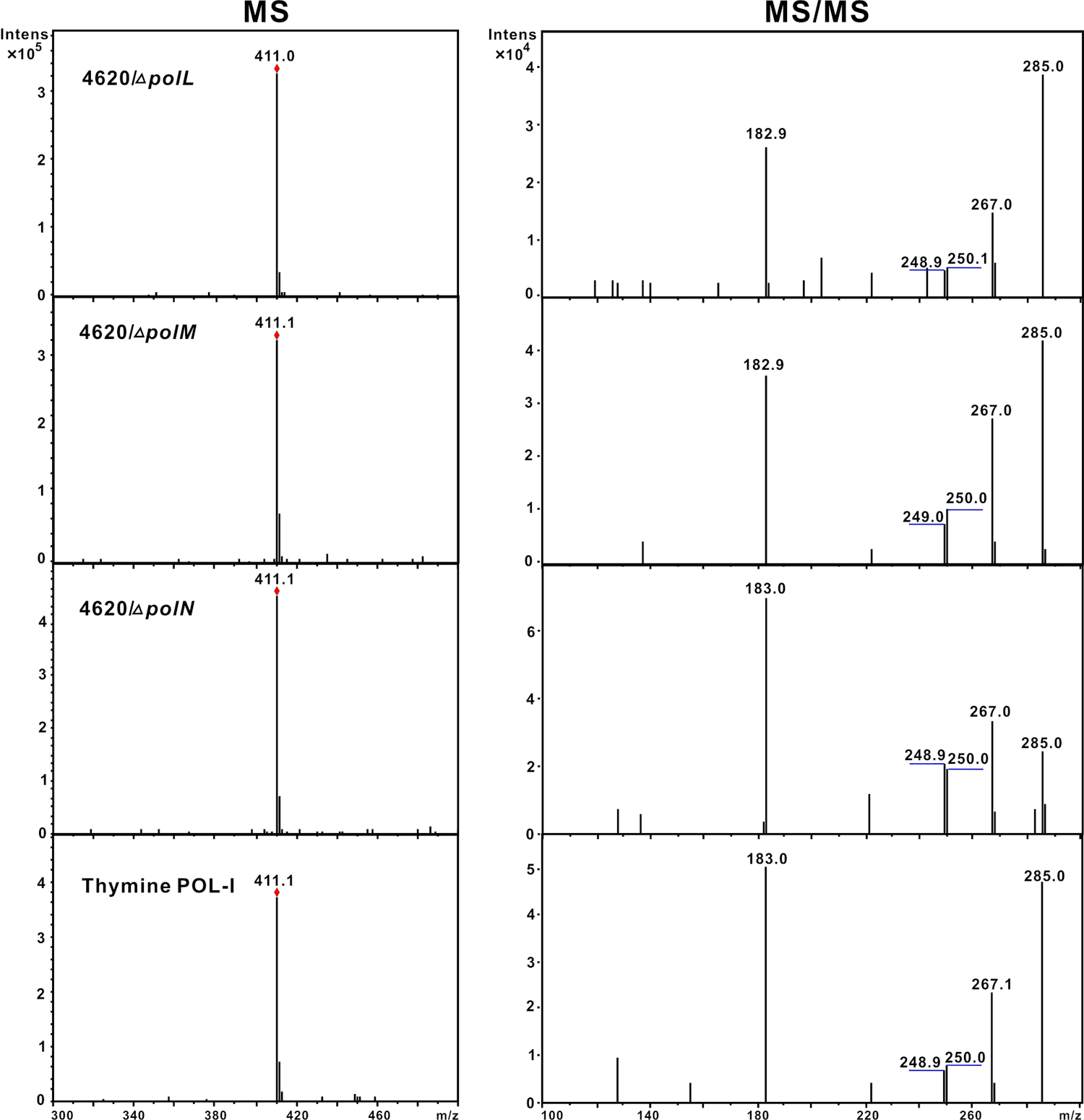
**

**Figure S2. MS and MS/MS analysis of the metabolites produced by pJTU4620 derivatives.** Left: MS analysis of the target metabolites produced by pJTU4620 derivatives. Right: MS/MS analysis of the target metabolites produced pJTU4620 derivatives.

**
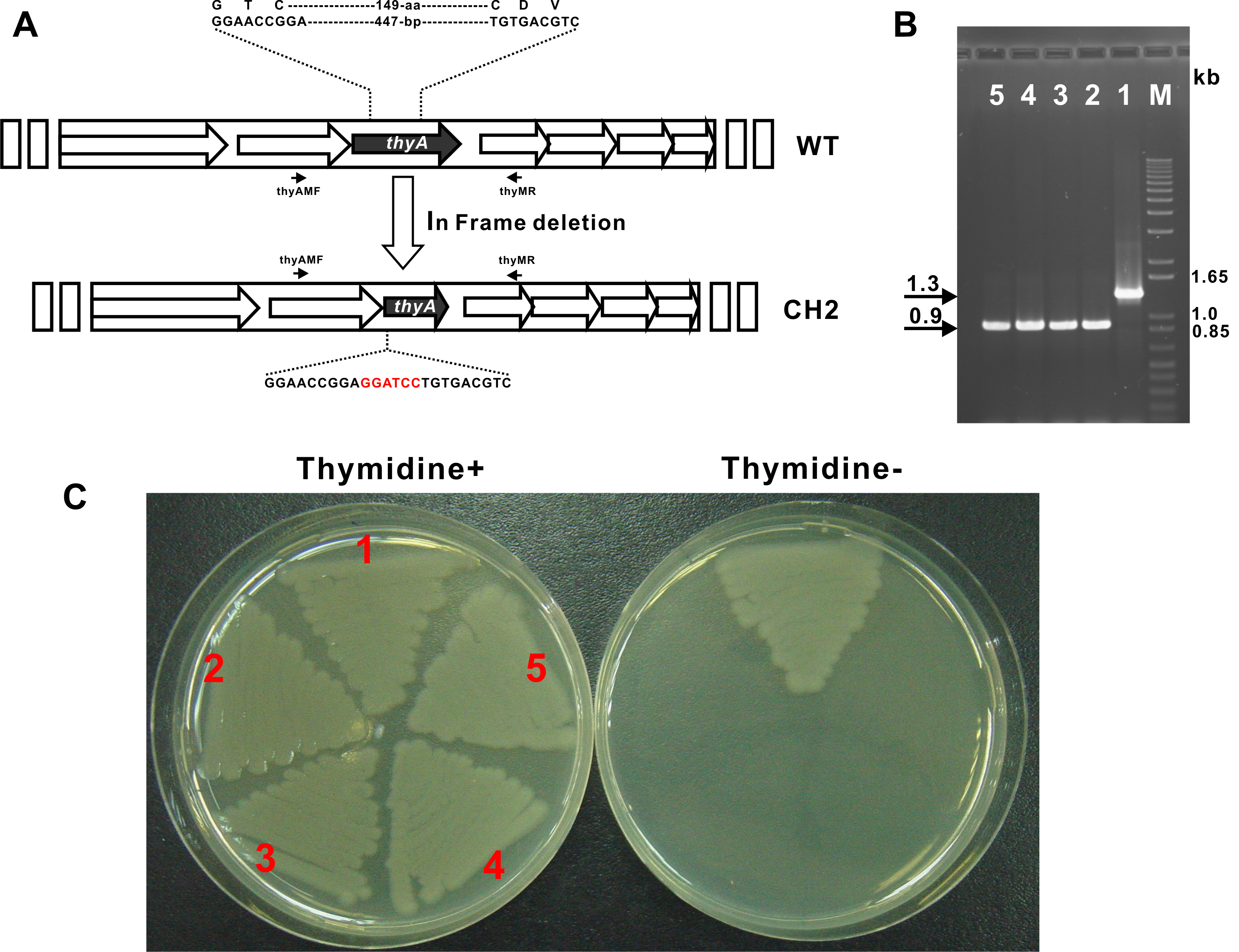
**

**Figure S3. Constrution of CH2 mutants and confirmation of its related biological phenotype.** (A). Representational map for the construction of CH2 mutant; (B). Identification of CH2 mutants, M: 1 kb plus ladder, 1: wild type of *E. coli* BL21(DE3), 2-5: CH2 mutants of *E. coli* BL21(DE3), as 477-bp fragment was deleted from *thyA*, the size of the PCR product for CH2 mutants was ca. 0.9-kb, while the wild type produces 1.3-kb PCR product; (C). Confirmation of the biological phenotype of CH2 mutants, 1: Wild type of *E. coli* BL21(DE3); 2-5: *E. coli* BL21(DE3) CH2 mutants, final concentration for thymidine used is ca. 50 μg/ml, and plates were incubated at 37℃ for 14 h.


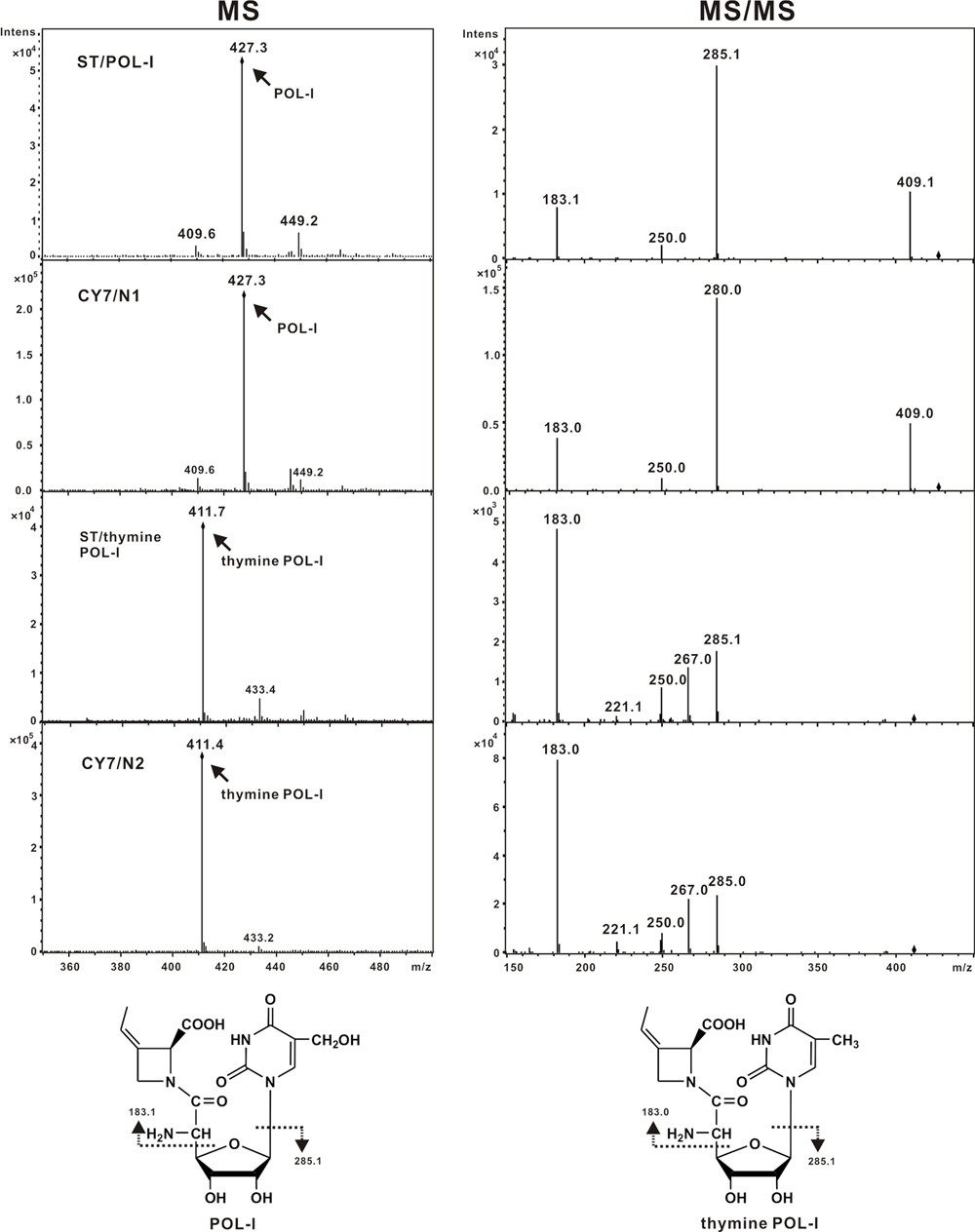


**Figure S4. LC/MS analysis of the metabolites produced by CY7 mutant.** ST/(Thymine) POL-I: (Thymine) POL-I authentic standard, ST/N1(N2): Novel compounds N1(N2) produced by CY7 mutant.

**
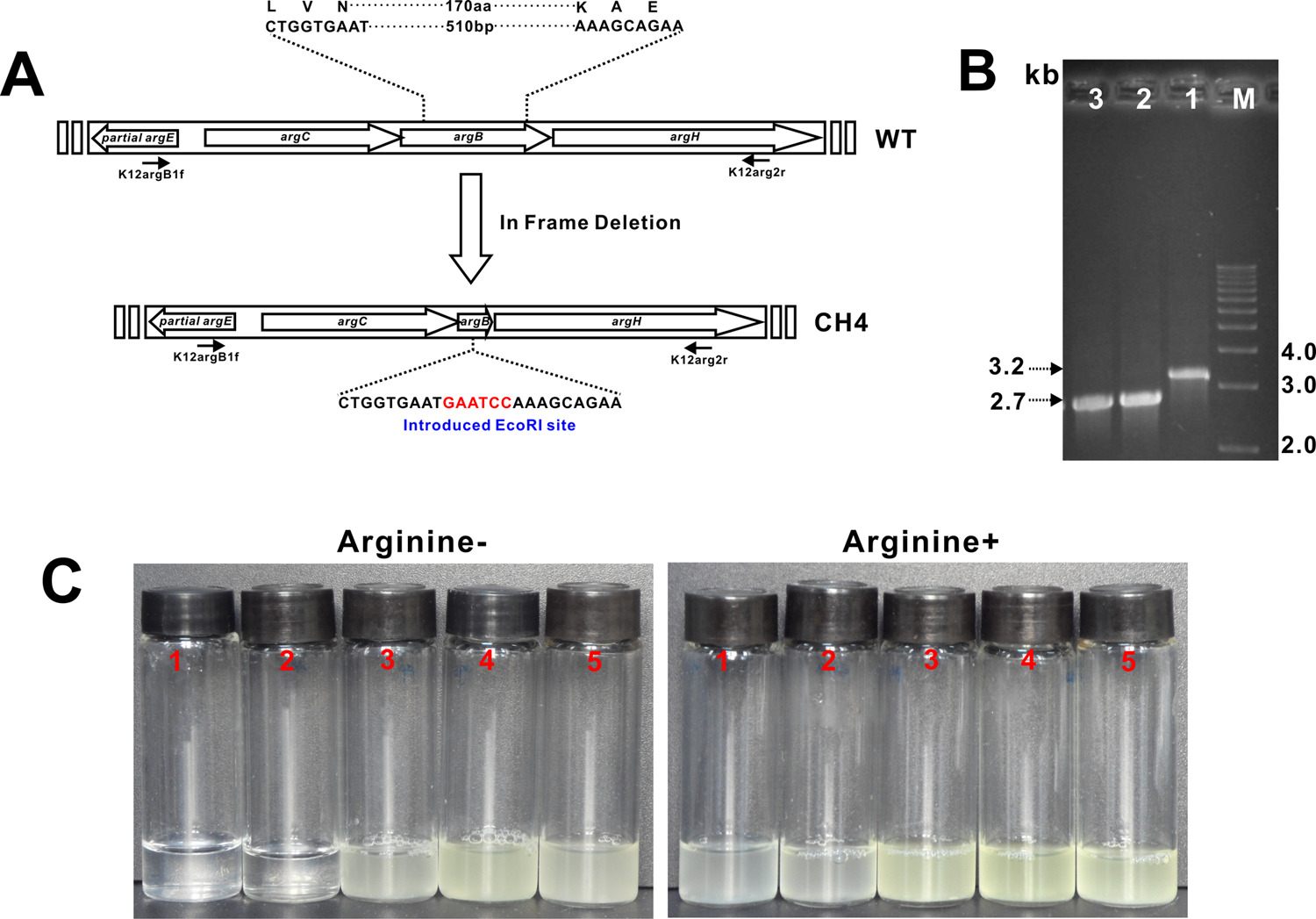
**

**Figure S5. Construction and Complementation of the CH4 Mutant. (**A). Representational map for construction of CH4 mutants; (B). PCR identification of CH4 mutants, M: 1 kb plus ladder, 1: Using genomic DNA of *E .coli* BL21(DE3) as template, 2-3: Using genomic DNA of CH4 mutants as template; (C). Minimal broth grown experiments for CH4 mutant and its complemented strains, 1: CH4 mutant, 2: CH4/pET28a, 3: CH4/pJTU2837, 4: CH4/pJTU2884, 5: *E. coli* BL21(DE3).


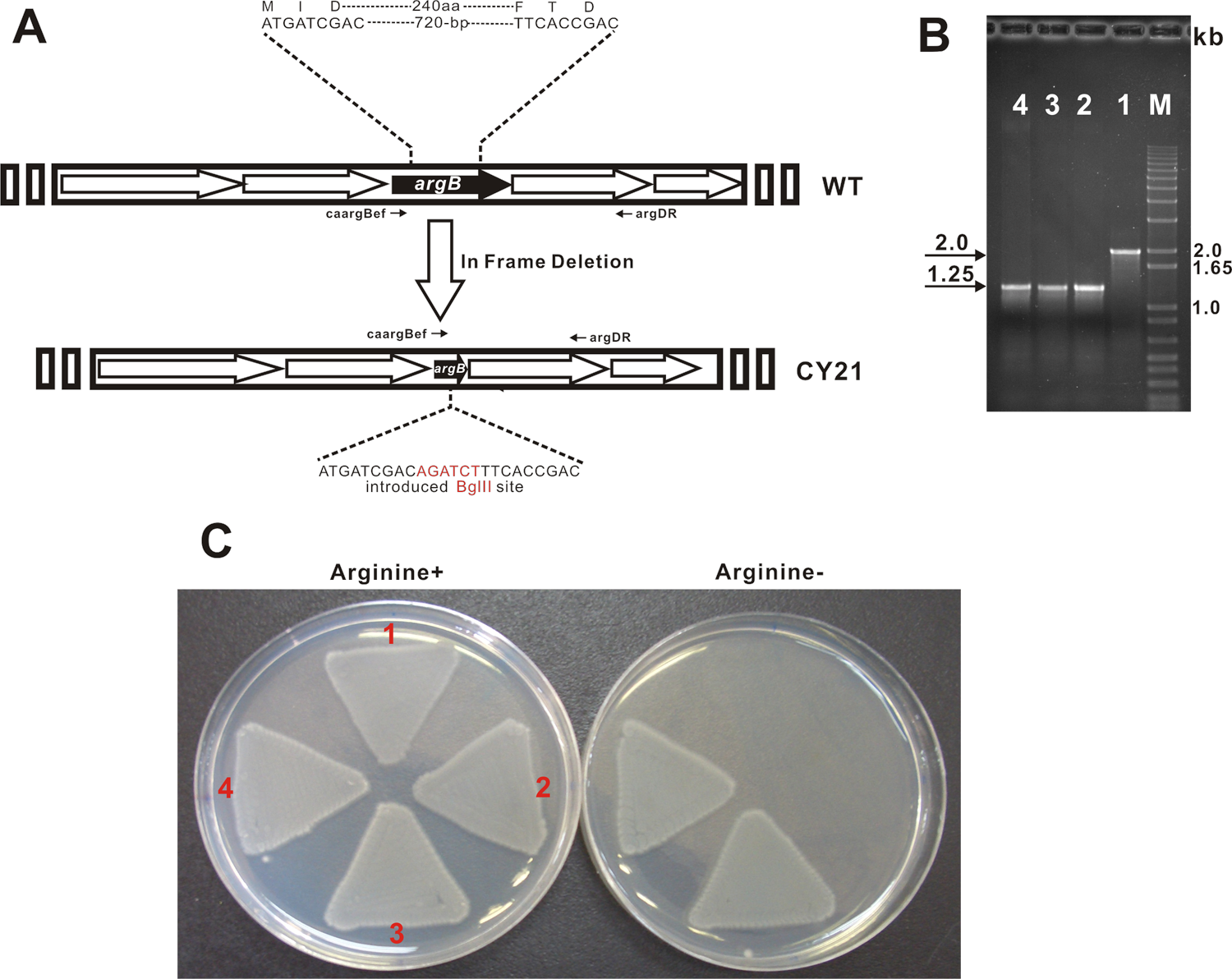


**Figure S6. Costruction and complementation of CY21 mutant. (**A). Representational map for construction of CY21 mutants; (B). PCR identification of CY21, M: 1 kb plus ladder, 1: Using genomic DNA of *S. cacaoi* WT as PCR template, CY21 2-4: Using genomic DNA of *S. cacaoi* CY21 mutants as PCR template; (C). Plate grown experiments for CY21 mutant and its complemented strain, 1: CY21 mutant, 2: CY21 mutant containing pJTU2170 as negative control, 3: CY21 mutant containing pJTU4713 (*argB* gene inserted into pJTU2170), 4: *S. cacaoi* wild type.

**References**

1. Suzuki S, Isono K, Nagatsu J, Mizutani T, Kawashima Y, Mizuno T: A New Antibiotic, Polyoxin A. *J Antibiot (Tokyo)* 1965, 18:131.

2. Kieser T, M. J. Bibb, K. F. Chater, M. J. Butter, and D. A. Hopwood.: Practical Streptomyces genetics. a laboratory manual. John Innes Foundation,. *Norwich, United Kingdom* 2000.

3. Paget MS, Chamberlin L, Atrih A, Foster SJ, Buttner MJ: Evidence that the extracytoplasmic function sigma factor sigmaE is required for normal cell wall structure in Streptomyces coelicolor A3(2). *J Bacteriol* 1999, 181:204-211.

4. Janssen GR, Bibb MJ: Derivatives of pUC18 that have BglII sites flanking a modified multiple cloning site and that retain the ability to identify recombinant clones by visual screening of Escherichia coli colonies. *Gene* 1993, 124:133-134.

5. Bierman M, Logan R, O'Brien K, Seno ET, Rao RN, Schoner BE: Plasmid cloning vectors for the conjugal transfer of DNA from Escherichia coli to Streptomyces spp. *Gene* 1992, 116:43-49.

6. Del Vecchio F, Petkovic H, Kendrew SG, Low L, Wilkinson B, Lill R, Cortes J, Rudd BA, Staunton J, Leadlay PF: Active-site residue, domain and module swaps in modular polyketide synthases. *J Ind Microbiol Biotechnol* 2003, 30:489-494.

7. Datsenko KA, Wanner BL: One-step inactivation of chromosomal genes in Escherichia coli K-12 using PCR products. *Proc Natl Acad Sci U S A* 2000, 97:6640-6645.

8. Gust B, Challis GL, Fowler K, Kieser T, Chater KF: PCR-targeted Streptomyces gene replacement identifies a protein domain needed for biosynthesis of the sesquiterpene soil odor geosmin. *Proc Natl Acad Sci U S A* 2003, 100:1541-1546.

9. Lalioti M, Heath J: A new method for generating point mutations in bacterial artificial chromosomes by homologous recombination in Escherichia coli. *Nucleic Acids Res* 2001, 29:E14.

10. Chen W, Huang T, He X, Meng Q, You D, Bai L, Li J, Wu M, Li R, Xie Z, et al: Characterization of the polyoxin biosynthetic gene cluster from Streptomyces cacaoi and engineered production of polyoxin H. *J Biol Chem* 2009, 284:10627-10638.
